# Supplementary material for: KRAS-mediated CCDC6 degradation drives xCT upregulation and ferroptosis evasion
Source: Apoptosis. 2026 Jul 14;31(8):194. doi: 10.1007/s10495-026-02400-4 (PMC13369686; doi:10.1007/s10495-026-02400-4)
Supplement: Supplementary file 1 — Supplementary Material 1 [file 10495_2026_2400_MOESM1_ESM.pdf]

Table S1: Mutational profiling of primary human colorectal cancer (CRC) samples.

MUT/WT	PIK3CA	KIT	PDGFRA	BRAF	EGFR	KRAS	NRAS	CCDC6 HSCORE	Quartile
**WT**	WT	WT	WT	WT	WT	WT	WT	0.6	Q1
**WT**	WT	WT	V824V	WT	WT	WT	WT	0.9	Q1
**WT**	WT	WT	WT	V600E	WT	WT	WT	1.0	Q1
**WT**	WT	WT	WT	V600E	WT	WT	WT	0.3	Q1
**WT**	E542K	WT	WT	WT	WT	WT	WT	0.5	Q1
**WT**	WT	WT	WT	WT	WT	WT	WT	1.0	Q1
**WT**	WT	WT	WT	WT	WT	WT	WT	1.2	Q1
**WT**						WT	WT	1.5	Q2
**WT**	WT	WT	WT	WT	WT	WT	WT	1.5	Q2
**WT**	WT	WT	WT	WT	WT	WT	WT	1.7	Q2
**WT**	WT	WT	WT	WT	WT	WT	WT	1.7	Q2
**WT**	WT	WT	WT	WT	WT	WT	WT	1.5	Q2
**WT**	WT	WT	WT	WT	WT	WT	WT	1.6	Q2
**WT**	WT	WT	WT	V600E	WT	WT	WT	1.7	Q2
**WT**	WT	WT	WT	WT	WT	WT	WT	1.3	Q2
**WT**	WT	WT	WT	WT	WT	WT	WT	1.3	Q2
**WT**	WT	WT	WT	WT	WT	WT	WT	1.5	Q2
**WT**	WT	WT	WT	WT	WT	WT	WT	2.2	Q3
**WT**	WT	WT	WT	WT	WT	WT	WT	1.8	Q3
**WT**	WT	WT	WT	WT	WT	WT	WT	2.0	Q3
**WT**	WT	WT	WT	WT	WT	WT	WT	2.0	Q3
**WT**	WT	WT	WT	WT	WT	WT	WT	1.8	Q3
**WT**	WT	WT	WT	WT	WT	WT	WT	2.2	Q3
**WT**	WT	WT	D594G	WT	WT	WT	WT	2.0	Q3
**WT**	WT	WT	WT	WT	WT	WT	WT	2.0	Q3
**WT**	WT	WT	V824V	WT	WT	WT	WT	1.8	Q3
**WT**	WT	M541L	WT	WT	WT	WT	WT	2.0	Q3
**WT**	WT	M541L	WT	WT	WT	WT	WT	2.0	Q3
**WT**						WT	WT	2.0	Q3
**WT**	WT	WT	V824V	WT	WT	WT	WT	2.6	Q4
**WT**	WT	WT	WT	WT	WT	WT	WT	2.8	Q4
**WT**	WT	WT	WT	WT	WT	WT	WT	3.0	Q4

**WT**	WT	M541L	V824V	V600E	WT	WT	WT	2.3	Q4
**WT**	Q546R	M541L	WT	WT	WT	WT	WT	2.3	Q4
**WT**	WT	WT	WT	WT	WT	WT	WT	2.4	Q4
**WT**	WT	WT	WT	WT	WT	WT	WT	2.5	Q4
**WT**	WT	WT	WT	WT	WT	WT	WT	2.55	Q4
**WT**	WT	WT	WT	WT	WT	WT	WT	3.0	Q4
**WT**	WT	WT	WT	WT	WT	WT	WT	3.0	Q4
**WT**	G1049R	WT	WT	WT	WT	WT	WT	2.5	Q4
**WT**	WT	M541L	WT	V600E	WT	WT	WT	3.0	Q4
**MUT**	WT	WT	WT	WT	WT	G13D		0.8	Q1
**MUT**	WT	M541L	WT	WT	WT	G13D		0.7	Q1
**MUT**	WT	M541L	WT	WT	WT	G13D		0.7	Q1
**MUT**	WT	WT	V824V	WT	WT	A146T		0	Q1
**MUT**	E545K	WT	WT	WT	WT	A146T		1.2	Q1
**MUT**	WT	WT	V824V	WT	WT	G12C		1.0	Q1
**MUT**	E542K	WT	V824V	WT	WT	G13D		0.5	Q1
**MUT**	WT	WT	V824V	WT	WT	G12D		0.6	Q1
**MUT**	WT	WT	WT	WT	WT	A146T		0.2	Q1
**MUT**	WT	WT	V828V	WT	WT	G12D		0.6	Q1
**MUT**	WT	WT	WT	WT	WT	G12D		1.0	Q1
**MUT**	WT	WT	WT	WT	WT	G12V		0	Q1
**MUT**	H1047L	WT	WT	WT	WT	G13D		1.2	Q1
**MUT**	E545K	M541L	WT	WT	WT	A146T		0.8	Q1
**MUT**	WT	WT	WT	WT	WT	G12D		1.2	Q1
**MUT**	WT	WT	WT	WT	WT		Q61K	1.2	Q1
**MUT**	WT	WT	WT	WT	WT	G13D		0.7	Q1
**MUT**	WT	WT	WT	WT	WT	G12S		0.1	Q1
**MUT**	WT	WT	WT	WT	WT	G13D		0.6	Q1
**MUT**	WT	WT	V824V	WT	WT	G12D		0.6	Q1
**MUT**	WT	WT	V824V	WT	WT	G13D		1.1	Q1
**MUT**	WT	WT	WT	WT	WT		Q61K	1.1	Q1
**MUT**	H1047R					G13D		0.7	Q1
**MUT**	/	/	/	/	/	G12D		1.1	Q1
**MUT**	WT	WT	WT	WT	WT	G13D		0	Q1

**MUT**	WT	WT	WT	WT	WT	G12C		1.1	Q1
**MUT**	WT	WT	WT	WT	WT	G12V		0.5	Q1
**MUT**	WT	WT	WT	WT	WT	G12A		1.6	Q2
**MUT**	WT	WT	V824V	WT	WT	L19F		1.4	Q2
**MUT**	WT	WT	V824V	WT	WT	G12S		1.3	Q2
**MUT**	WT	M541L	WT	WT	WT	Q61K		1.7	Q2
**MUT**	WT	WT	WT	WT	WT		Q61K	1.55	Q2
**MUT**	H1047R	WT	WT	WT	WT	G12V		1.6	Q2
**MUT**	E542K		V824V			G12D		1.7	Q2
**MUT**	WT	M541L	WT	WT	WT	G12V		1.7	Q2
**MUT**	WT	WT	WT	WT	WT	G12D		1.3	Q2
**MUT**	WT	WT	WT	WT	WT	G12V		2.1	Q3
**MUT**	WT	WT	WT	WT	WT	G13D		2.0	Q3
**MUT**	WT	WT	WT	WT	WT	Q61H		2.0	Q3
**MUT**	WT	WT	V824V	WT	WT	G12D		2.2	Q3
**MUT**	WT	WT	WT	WT	WT	G12D		2.2	Q3
**MUT**	WT	WT	WT	WT	WT	G12D		2.0	Q3
**MUT**	WT	WT	WT	WT	WT		Q61K	2.0	Q3
**MUT**	/	/	/	/	/	G12D		2.2	Q3
**MUT**	/	/	/	/	/	Q61H		2.0	Q3
**MUT**	WT	WT	WT	WT	WT	Q61H		1.8	Q3
**MUT**	/	/	/	/	/	G13D		2.0	Q3
**MUT**	/	/	/	/	/	G12D		2.0	Q3
**MUT**	WT	WT	WT	WT	WT	G12V		2.0	Q3
**MUT**	WT	WT	WT	WT	WT	G12V		3.0	Q4
**MUT**	WT	WT	WT	G469R	WT		G12V	2.3	Q4
**MUT**	E545K	M541L	WT	WT	WT	G12D		3.0	Q4
**MUT**	WT	WT	WT	WT	WT	G13D		2.6	Q4
**MUT**	WT	WT	V824V	WT	WT	G12D		2.6	Q4
**MUT**	WT	WT	WT	WT	WT	G12V		2.9	Q4
**MUT**	WT	WT	WT	WT	WT	G12D		3.0	Q4
**MUT**	WT	WT	WT	V600E	WT	G12D		3.0	Q4
**MUT**	E542K		V824V			G12D		3.0	Q4
**MUT**	WT	WT	WT	WT	WT	G12V		2.6	Q4

**MUT**	E545K					G12D		2.6	Q4

Table S1. Mutational profiling of primary human colorectal cancer (CRC) samples.
A total of 101 primary CRC specimens were screened via next-generation sequencing (NGS) for RAS mutational status. Among these, 60 samples harbored RAS mutations, 55 KRAS and 5 NRAS (MUT), while 41 samples were confirmed as RAS wild type (WT). The table details the distribution of specific KRAS and NRAS isoforms, as well as additional co-occurring mutations identified across both WT and mutant cohorts.
