# Supplementary material for: KRAS-mediated CCDC6 degradation drives xCT upregulation and ferroptosis evasion
Source: Apoptosis. 2026 Jul 14;31(8):194. doi: 10.1007/s10495-026-02400-4 (PMC13369686; doi:10.1007/s10495-026-02400-4)

Figure S1

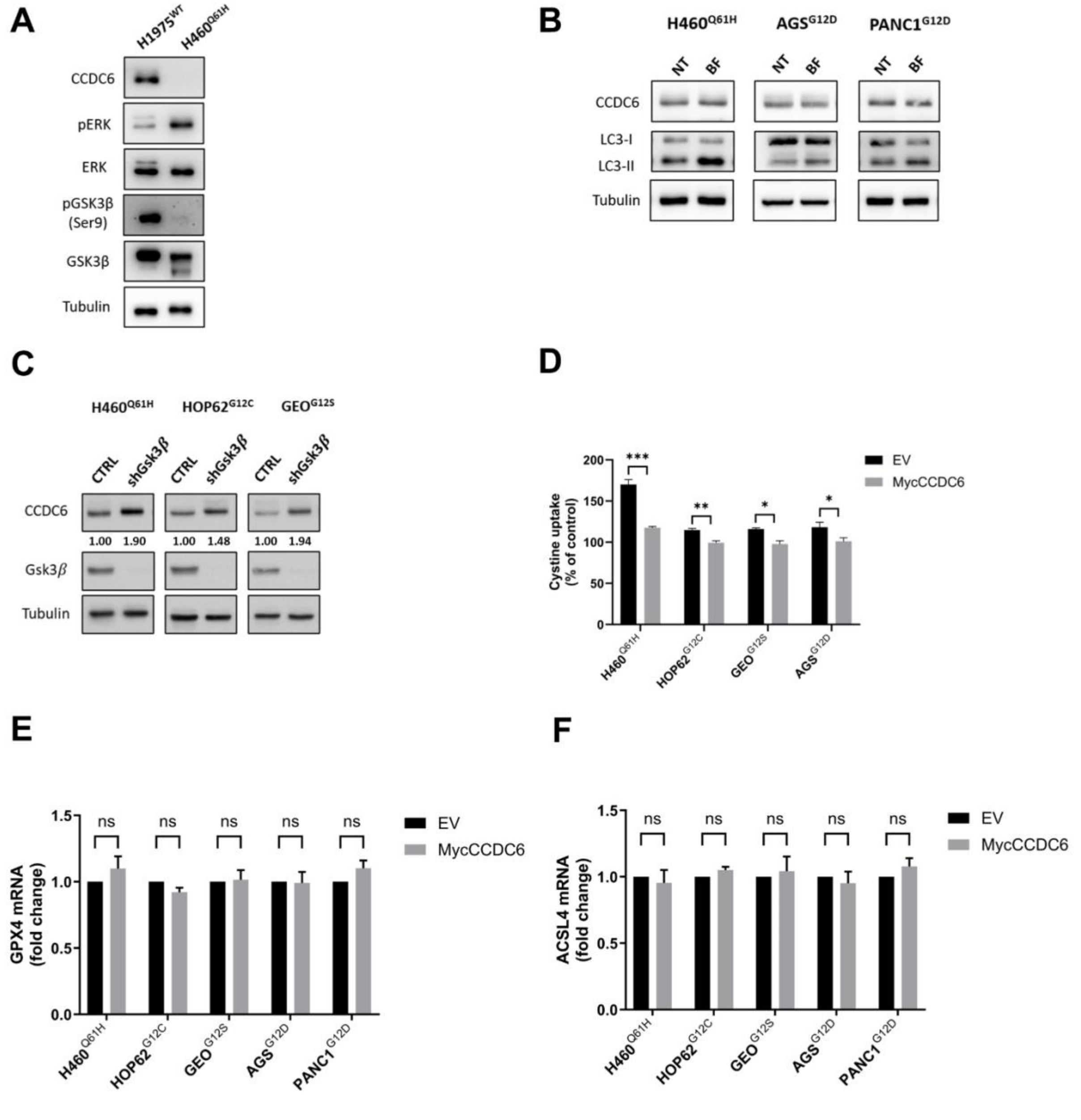

Figure S1.

A-C: Regulation of CCDC6 stability by GSK3β and lysosomal pathways;

(A)Western blot analysis of CCDC6, pERK and pGSK3β (Ser9) in H1975 and H460 cells. Anti-tubulin immunoblot is shown as loading control.

(B) Western blot analysis of CCDC6 in H460, AGS and PANC1 cells treated with Bafilomycin A1 (BF) [100nM] for 2h. Anti-LC3 immunoblots demonstrate the efficacy of the treatment. Anti-tubulin immunoblots are shown as loading control.

(C) Western blot analysis of CCDC6 expression in H460, HOP62 and GEO cells upon transient transfection with shGSK3β. Anti-shGSK3β and anti-tubulin immunoblots are shown as transfection and loading control, respectively.

D-F: Impact of CCDC6 re-expression on cystine uptake and on GPX4 and ACSL4 expression

(D) Cystine uptake of H460, HOP62, GEO and AGS cells transfected with MycCCDC6 or empty vector (EV) and treated with Diethyl Malonate (DEM) [1mM] for 3h was assessed by a BioTracker Cystine-FITC Live Cell Dye followed by flow cytometry analysis.

(E-F) Relative expression of GPX4 (E) and ACSL4 (F) assessed by qPCR in H460, HOP62, GEO, AGS and PANC1 cells upon transient transfection of MycCCDC6 or empty vector (EV), as a control.

Figure S2

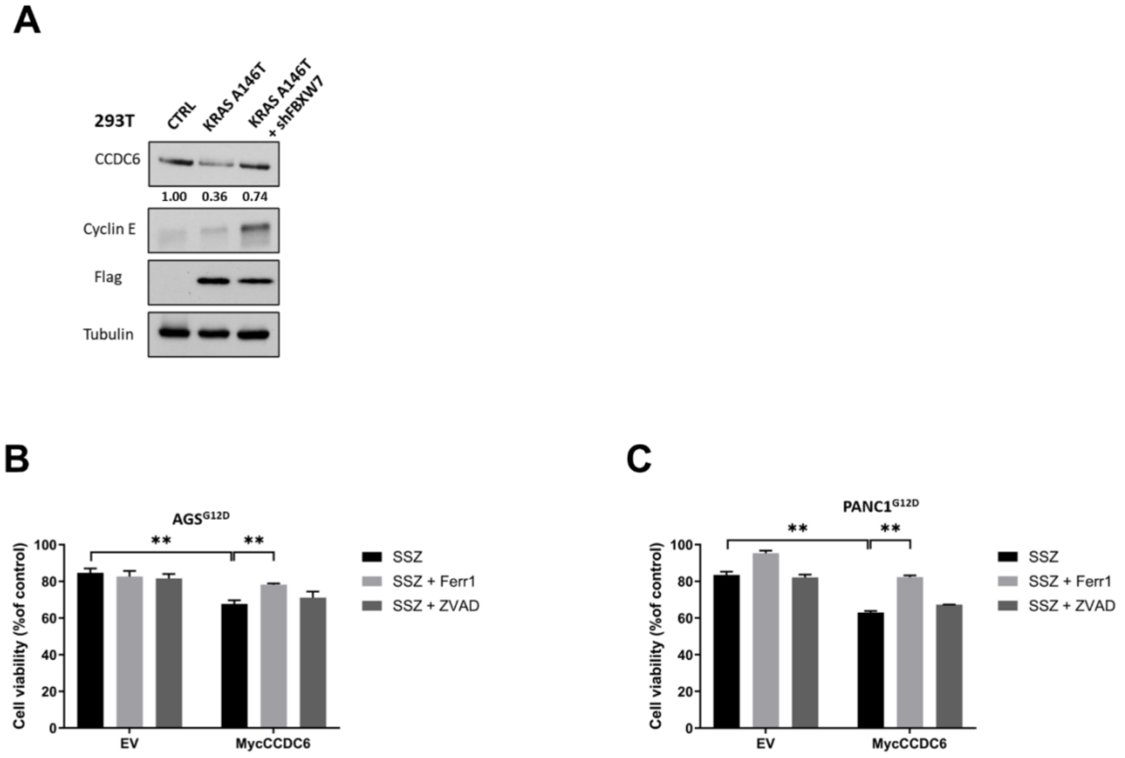

Figure S2.

A: FBXW7 involvement in KRAS-mediated CCDC6 degradation.

(A) Western blot analysis of CCDC6 expression in 293T cells transfected with KRAS isoform A146T or empty vector (CTRL), in presence or absence of shFBXW7. Expression levels of cyclin E are shown as a representative substrate of FBXW7. Anti-Flag immunoblots was performed to confirm the expression of Flag-KRAS mutant plasmid. Anti-tubulin is shown as loading control.

B-C: Functional analysis of cell death pathways upon MycCCDC6 expression.

(B-C) Cell viability was assessed in AGS (B) and PANC1 (C) cells transfected with MycCCDC6 or empty vector (EV) and treated with Sulfasalazine (SSZ) (1mM AGS, 400µM PANC1) alone or combined with 10µM Z-VAD-fmk (ZVAD) or 10µM ferrostatin-1 (Ferr1) for 24h.

Figure S3

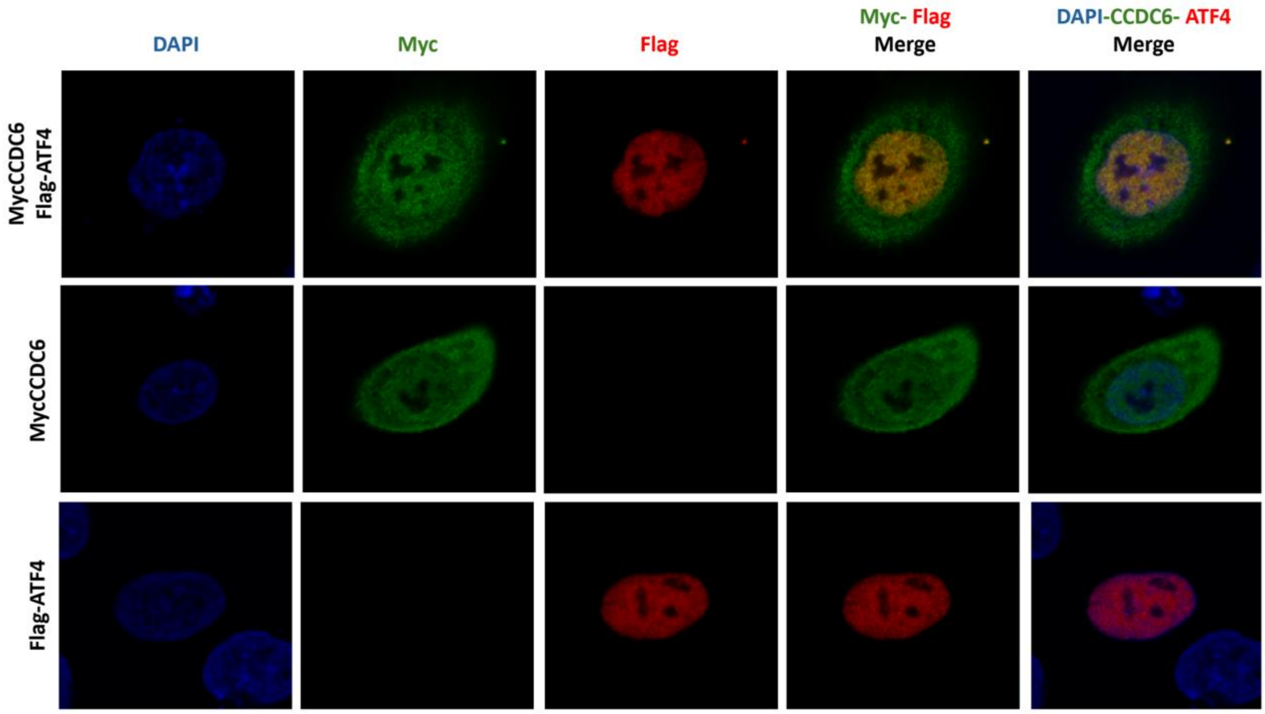

Figure S3.

Co-localization of CCDC6 and ATF4 in KRAS mutated gastric cancer cells.

Representative immunofluorescence images showing the nuclear co-localization of ATF4 and CCDC6 in AGS cells following transient co-transfection with Myc-CCDC6 and Flag-ATF4 plasmids. Single transfections of Myc-CCDC6 and Flag-ATF4 are shown as controls. DAPI and merged images are also included as indicated.

Figure S4

A

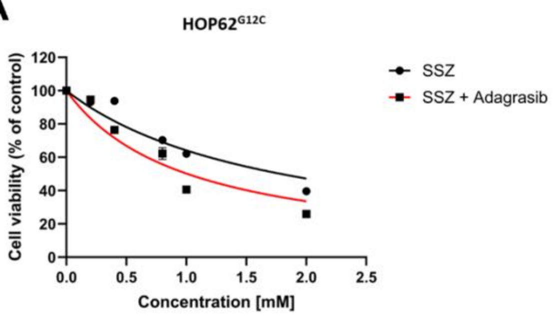

Drug sensitivity (IC50)<br>HOP62<br>Adagrasib [2µM]	-	+	Combination index (CI)	Dose Reduction Index (DRI)
SSZ [mM]	1.79 +/- 0.15	1.01 +/- 0.09	0.78	2.04

B

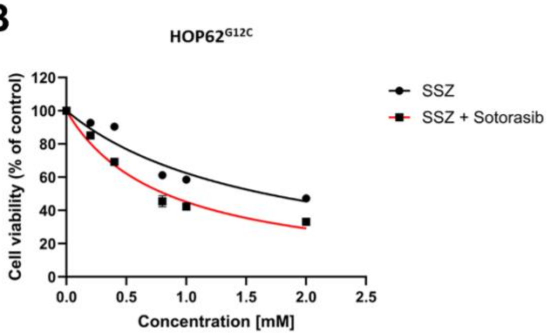

Drug sensitivity (IC50)<br>HOP62<br>Sotorasib [40µM]	-	+	Combination index (CI)	Dose Reduction Index (DRI)
SSZ [mM]	1.67 +/- 0.11	0.82 +/- 0.04	0.68	2.08

C

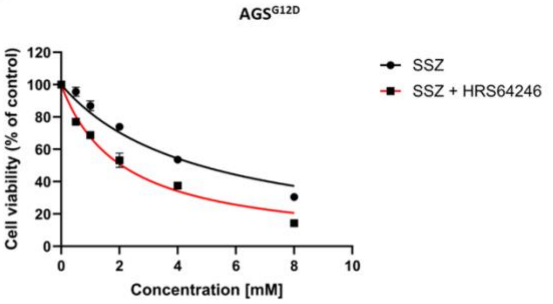

Drug sensitivity (IC50)<br>AGS<br>HRS-4642 [6nM]	-	+	Combination index (CI)	Dose Reduction Index (DRI)
SSZ [mM]	4.76 +/- 0.32	2.08 +/- 0.12	0.58	1.57

D

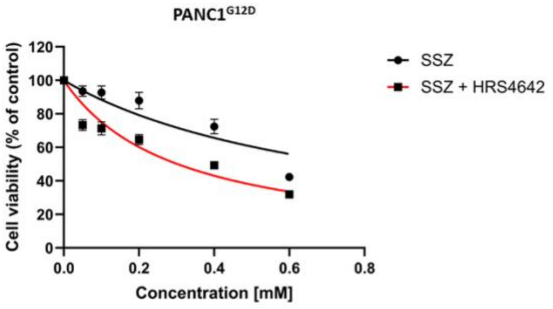

Drug sensitivity (IC50)<br>PANC1<br>HRS-4642 [5µM]	-	+	Combination index (CI)	Dose Reduction Index (DRI)
SSZ [mM]	0.76 +/- 0.10	0.3 +/- 0.03	0.72	1.89

Figure S4.

KRAS inhibitors and Sulfasalazine effect on cell viability.

(A-D) The synergistic effect of combining Sulfasalazine (SSZ) with KRAS inhibitors was evaluated using dose-response curves. IC50 values for Sulfasalazine (SSZ) were determined in the absence or presence of a fixed concentration of KRASG12C inhibitors (Adagrasib or Sotorasib) in HOP62 cells (A, B) and the KRASG12D inhibitor (HRS4642) in AGS (C) and PANC1 (D) cells for 24h. The synergy was confirmed by calculating the Combination Index (CI) and the Dose Reduction Index (DRI) from the same curves.

Figure S5

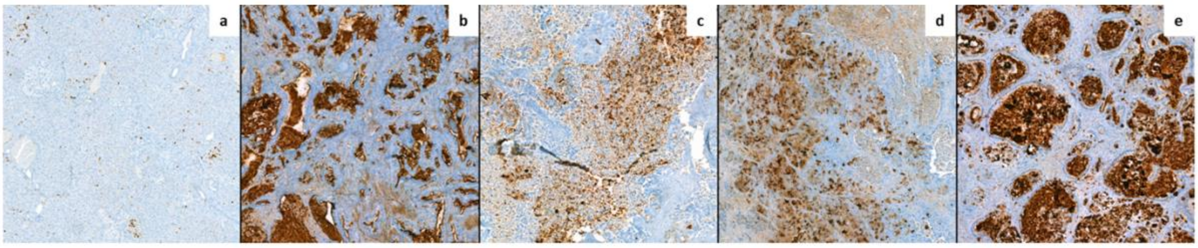

Figure S5: Immunohistochemical (IHC) analysis of pMAPK expression in Patient-Derived Xenografts (PDX) of PDAC.

Representative images showing pMAPK protein levels in normal pancreatic ductal tissue (a) and in PDX samples with different KRAS status: KRAS wild type (b), KRAS G12D (c), KRAS G12D (d), KRAS G12R (e). Scale bar = 100 μm.

Figure S6

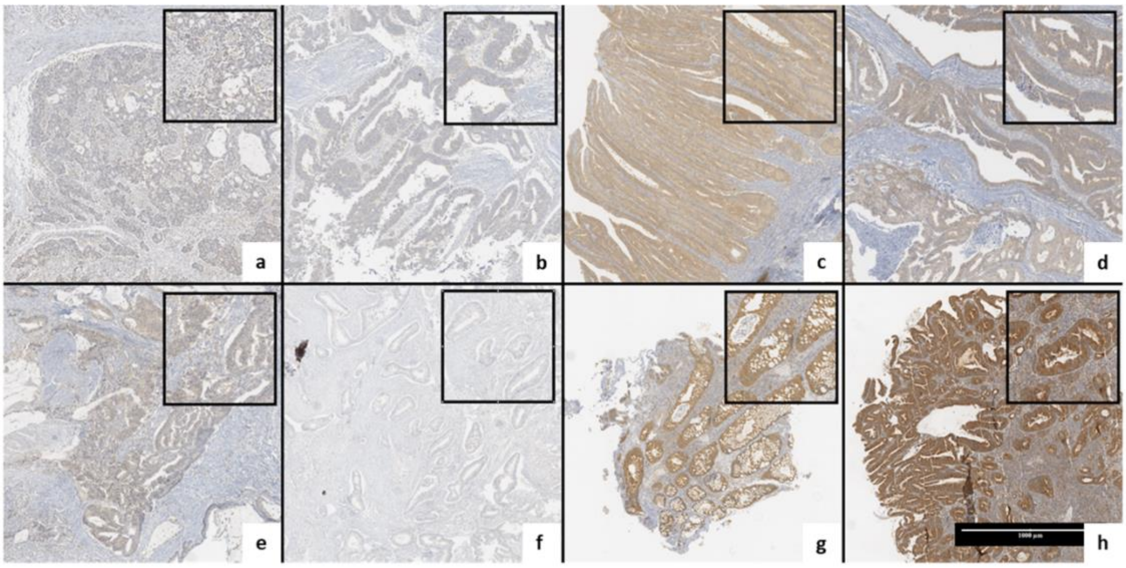

Figure S6: Immunohistochemical (IHC) analysis of CCDC6 expression in primary colorectal carcinoma (CRC) samples.
Representative images showing CCDC6 protein levels in KRAS wild-type (WT) cases with Absent/Low (a, b) or High (c, d) CCDC6 expression, and in KRAS mutant (MUT) cases with Absent/Low (e, f) or High (g, h) CCDC6 expression. Scale bar = 1000 µm.

Figure S7

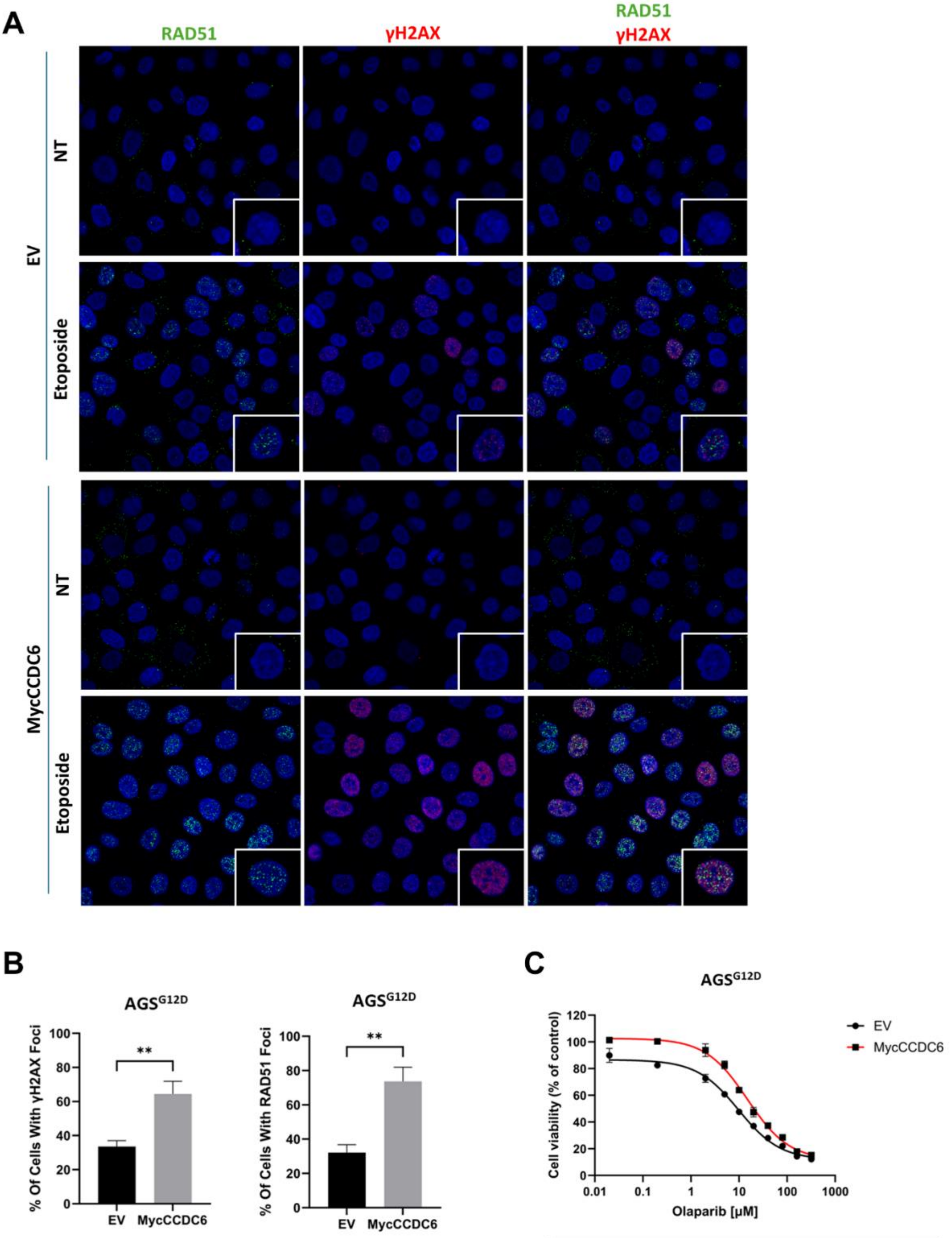

Drug sensitivity (IC50)	
AGS	Olaparib [μM]
EV	10.15 +/- 1.22
MycCCDC6	22.95 +/- 1.56

Figure S7: Re-expression of CCDC6 restores HR repair efficiency and induces PARP inhibitor resistance in AGS^{G12D} cells.

(A) Representative immunofluorescence images of AGS cells transiently transfected with MycCCDC6 or empty vector (EV) as a control. Cells were either left untreated (NT) or treated with Etoposide [10 μM] for 4h (to induce DNA damage) and stained for γH2AX (red), RAD51 (green), and DAPI (blue) to visualize DNA damage foci formation and the nucleus, respectively.

(B) Bar graphs represent the percentage of AGS cells with more than 5 foci. Data are reported as mean ± SEM of 3 independent repeats. Statistical significance was verified by 2-tailed Student's t-test (** $p < 0.01$).

(C) Dose-response curves were generated to assess the effect of CCDC6 re-expression on AGS cell viability in the presence of increasing concentrations of the PARP inhibitor Olaparib for 96h. MycCCDC6 re-expression was compared to an empty vector (EV) control. The IC50 values were calculated for each condition. The table (bottom) summarizes the calculated IC50 values ± SD.

Figure S8

Original Western Blot Relative to Figure 1A

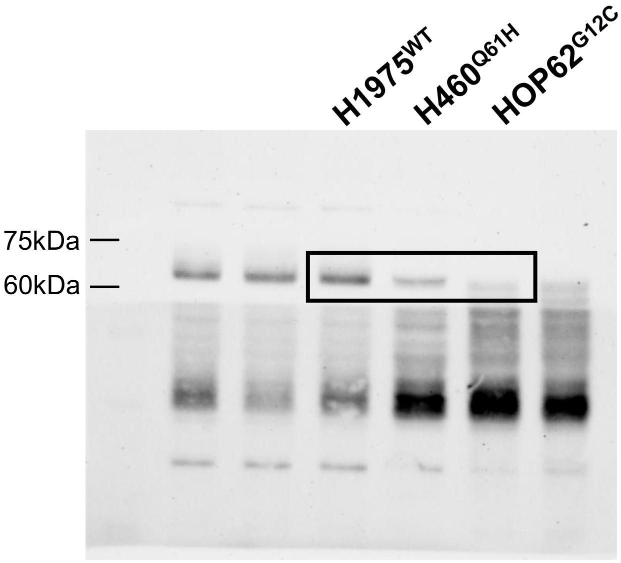

CCDC6

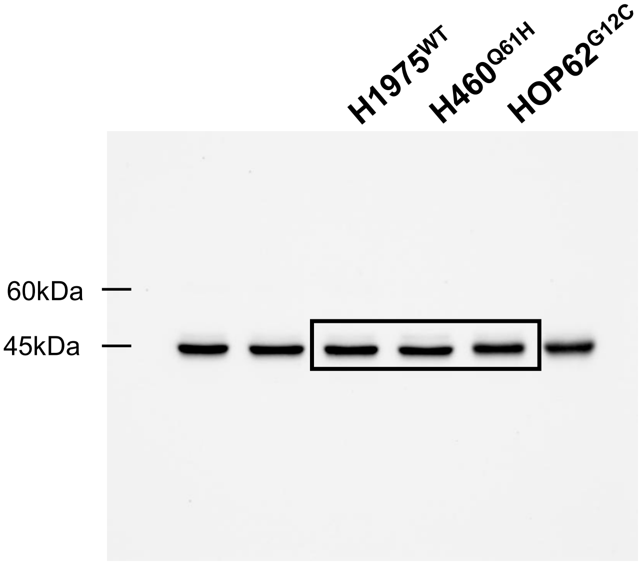

Tubulin

Original Western Blot Relative to Figure 1C

H460^{Q61H}

MG132 - +

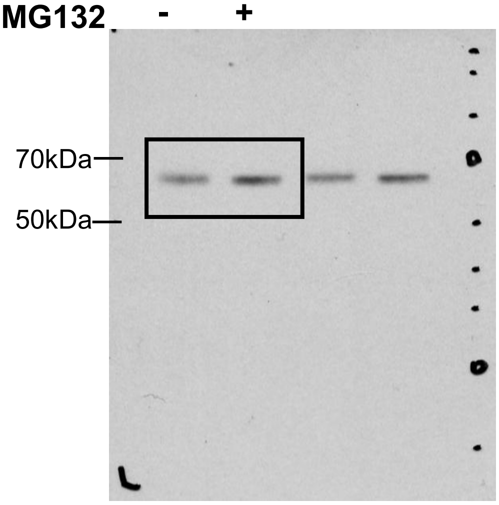

CCDC6

H460^{Q61H}

MG132 - +

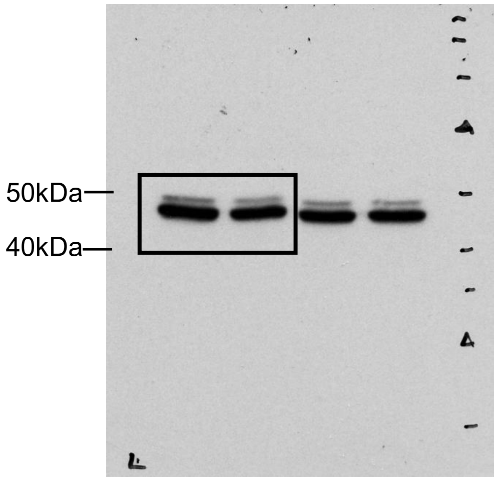

Tubulin

HOP62^{G12C}

MG132 - +

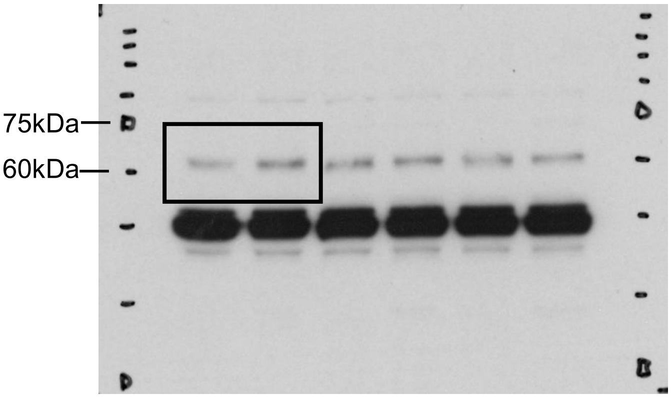

CCDC6

HOP62^{G12C}

MG132 - +

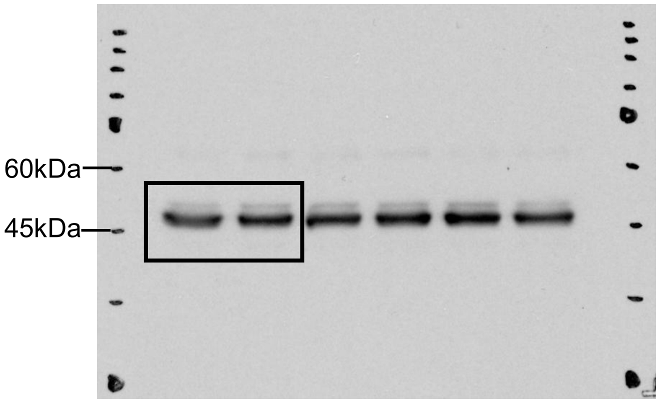

Tubulin

GEO^{G12s}

MG132 - +

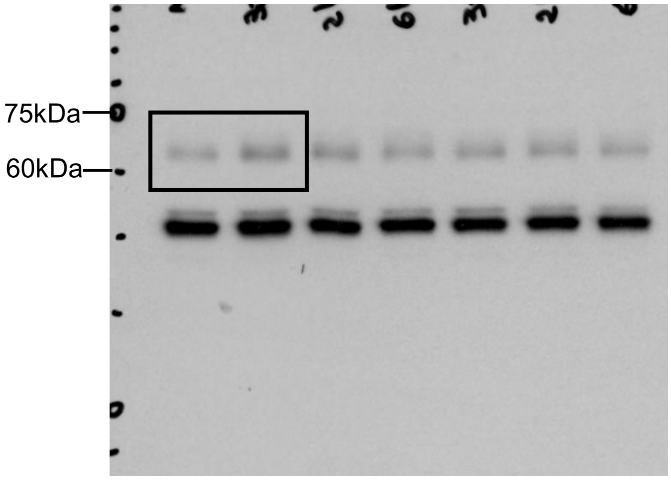

CCDC6

GEO^{G12s}

MG132 - +

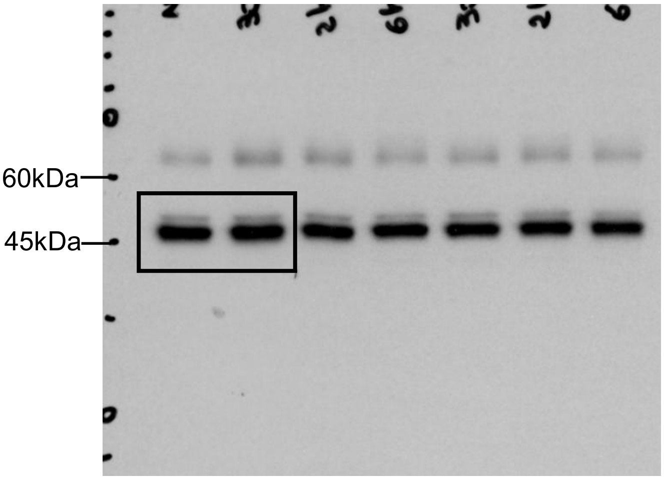

Tubulin

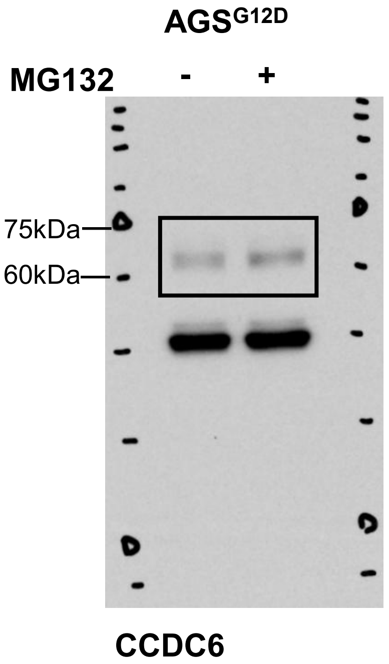
AGS^{G12D}
MG132 - +
75kDa
60kDa
CCDC6

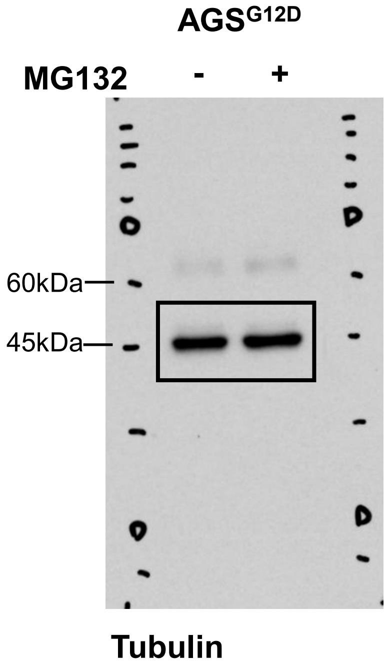
AGS^{G12D}
MG132 - +
60kDa
45kDa
Tubulin

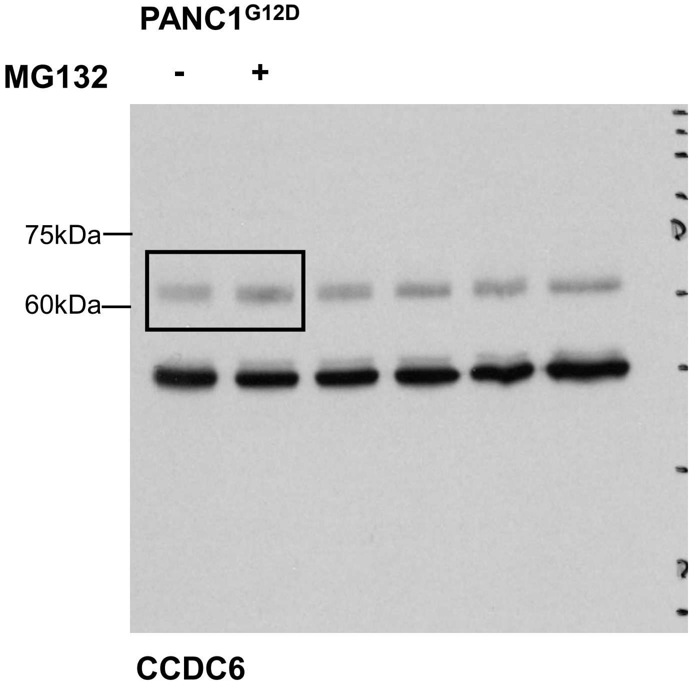
PANC1^{G12D}
MG132 - +
75kDa
60kDa
CCDC6

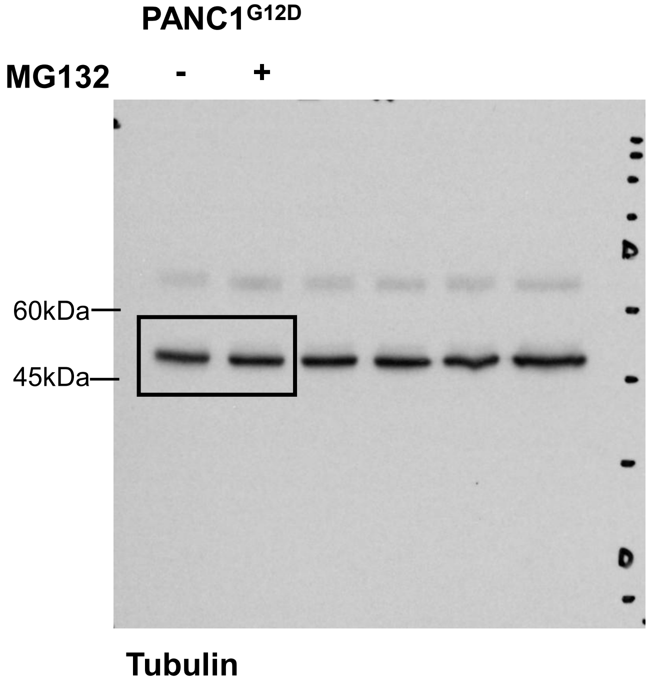
PANC1^{G12D}
MG132 - +
60kDa
45kDa
Tubulin

Original Western Blot Relative to Figure 1D

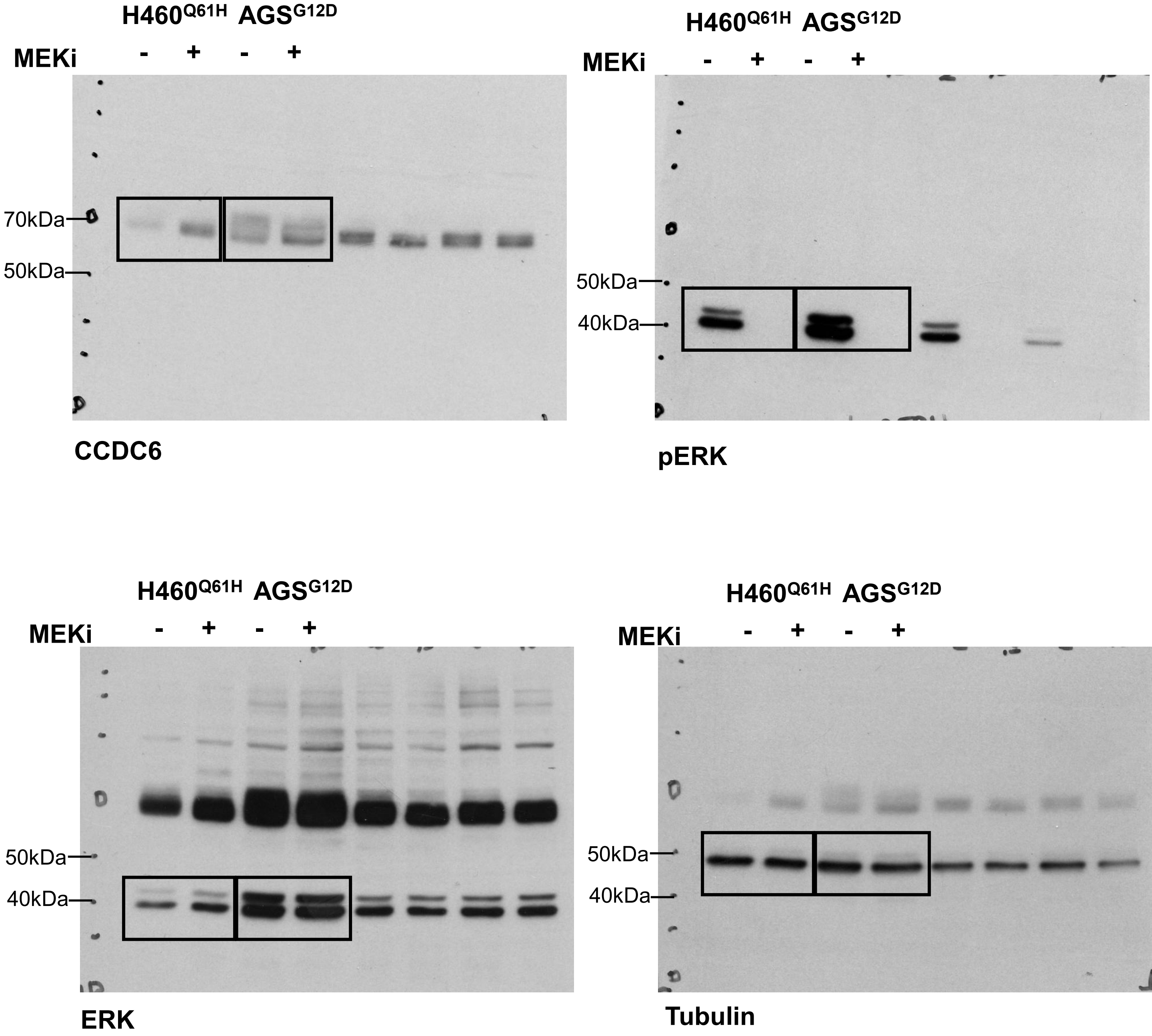

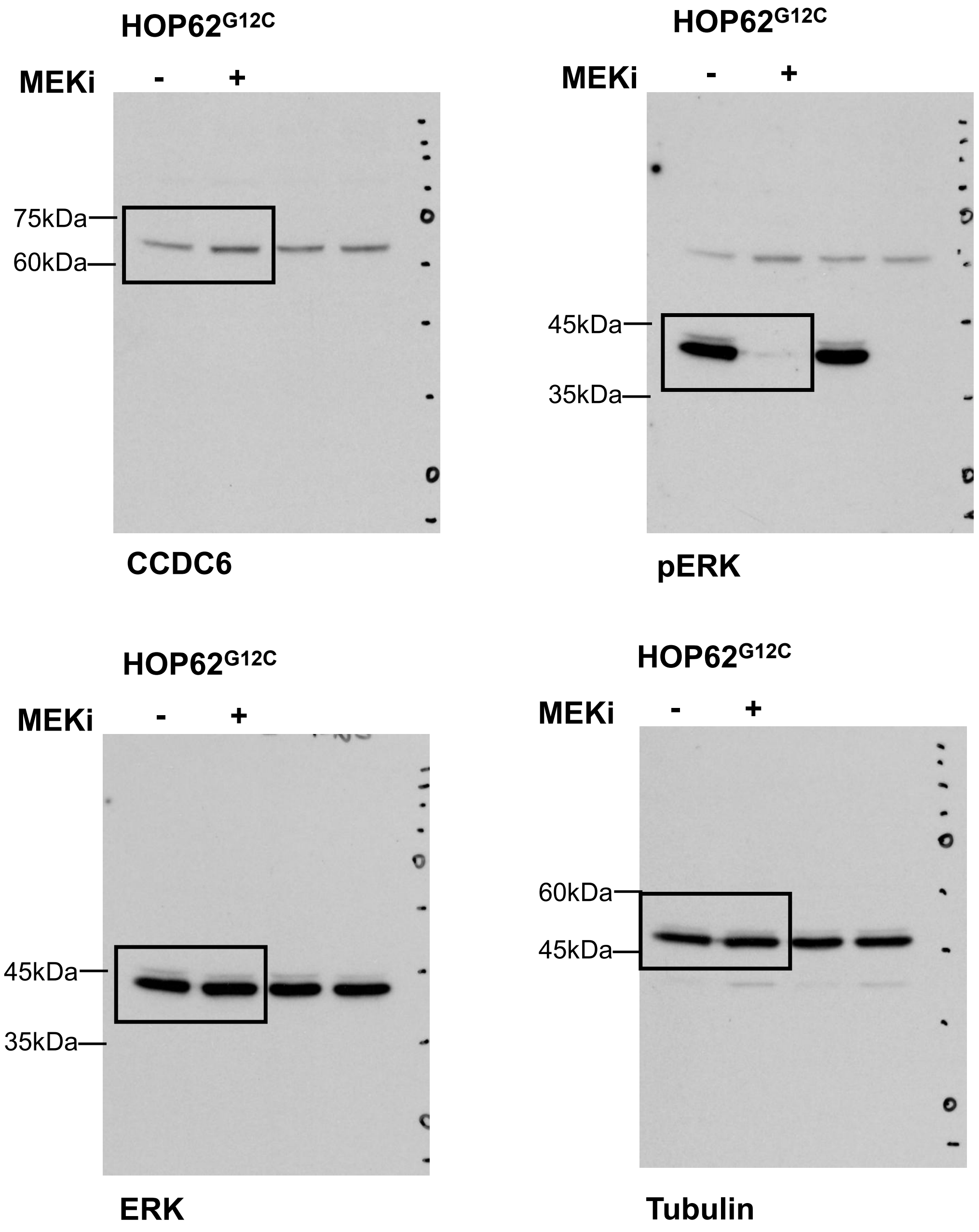

HOP62^{G12C}
MEKi - +
75kDa
60kDa
CCDC6
HOP62^{G12C}
MEKi - +
45kDa
35kDa
pERK
HOP62^{G12C}
MEKi - +
45kDa
35kDa
ERK
HOP62^{G12C}
MEKi - +
60kDa
45kDa
Tubulin

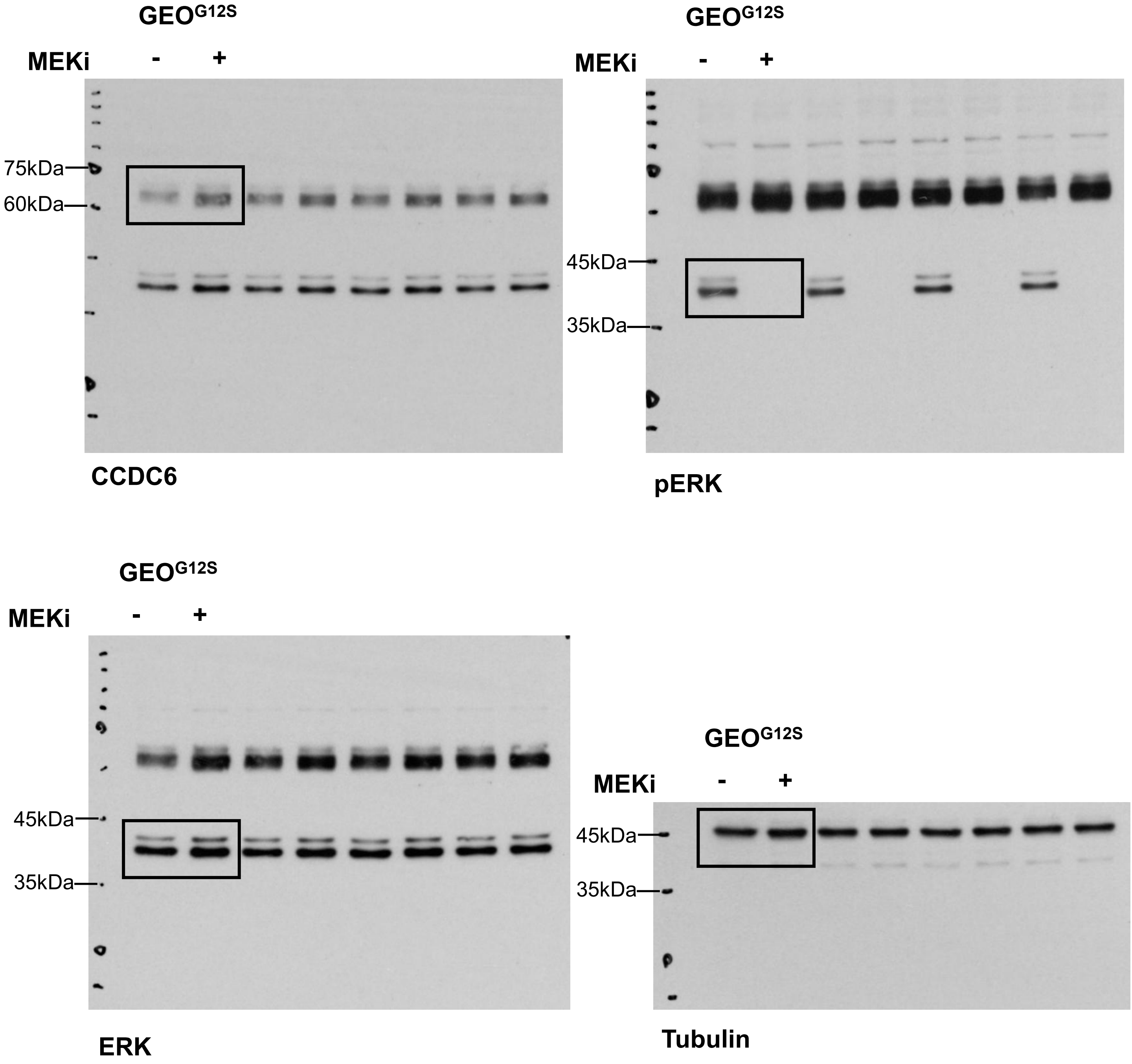
GEO^{G12S}
MEKi - +
75kDa
60kDa
CCDC6
GEO^{G12S}
MEKi - +
45kDa
35kDa
pERK
GEO^{G12S}
MEKi - +
45kDa
35kDa
ERK
GEO^{G12S}
MEKi - +
45kDa
35kDa
Tubulin

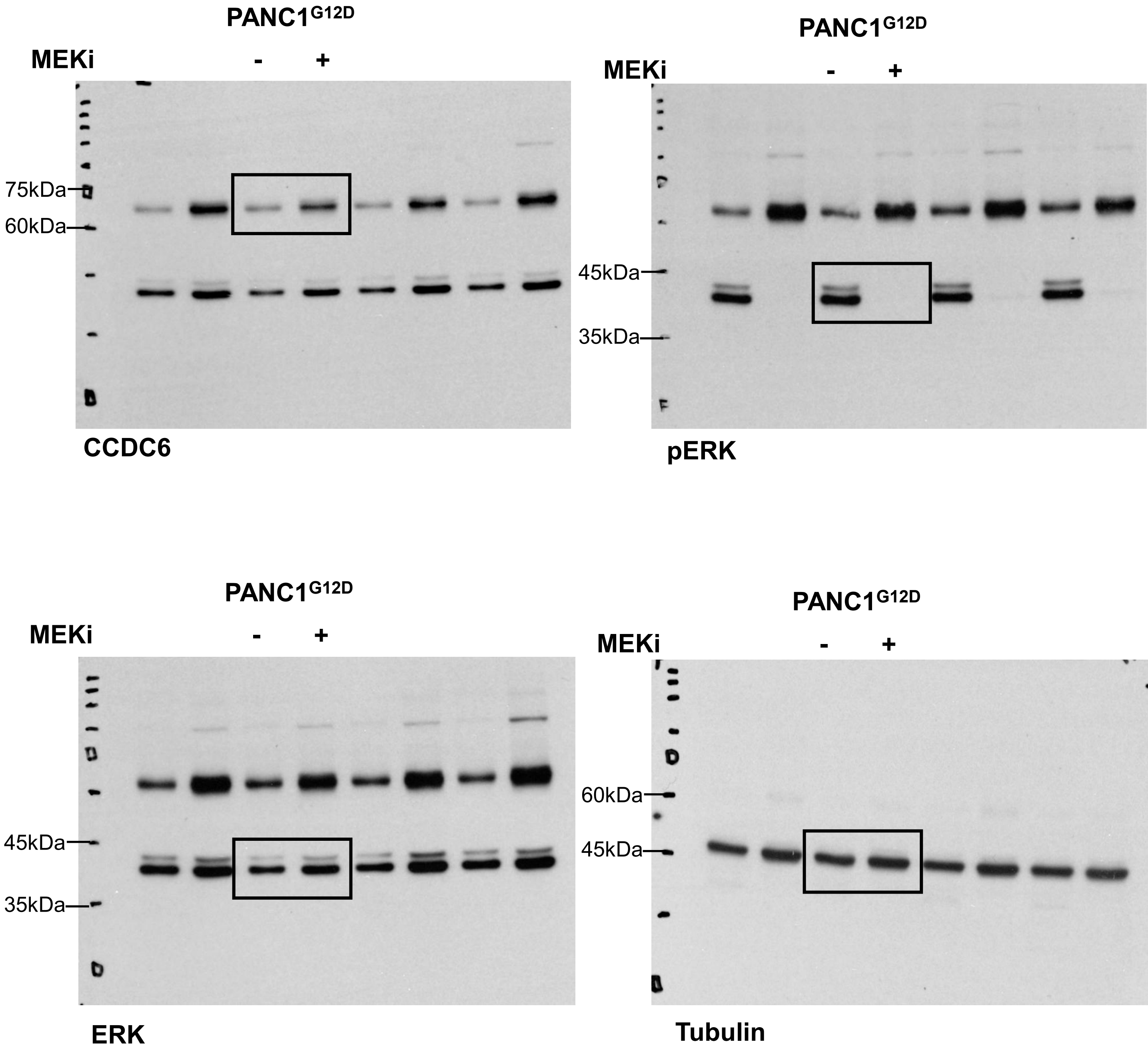

PANC1G12D
MEKi
-
+
75kDa
60kDa
CCDC6
PANC1G12D
MEKi
-
+
45kDa
35kDa
pERK
PANC1G12D
MEKi
-
+
45kDa
35kDa
ERK
PANC1G12D
MEKi
-
+
60kDa
45kDa
Tubulin

Original Western Blot Relative to Figure 1E

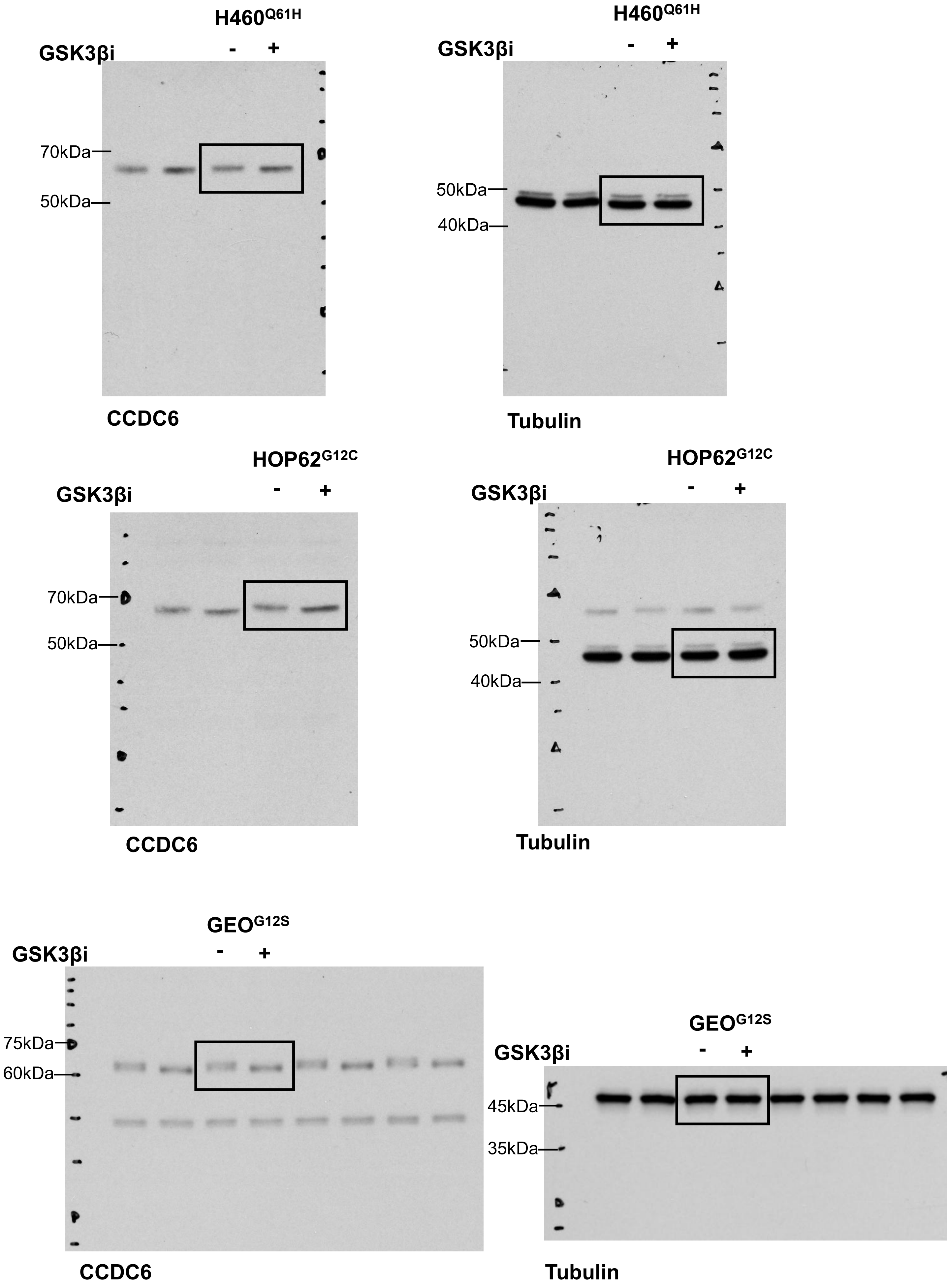

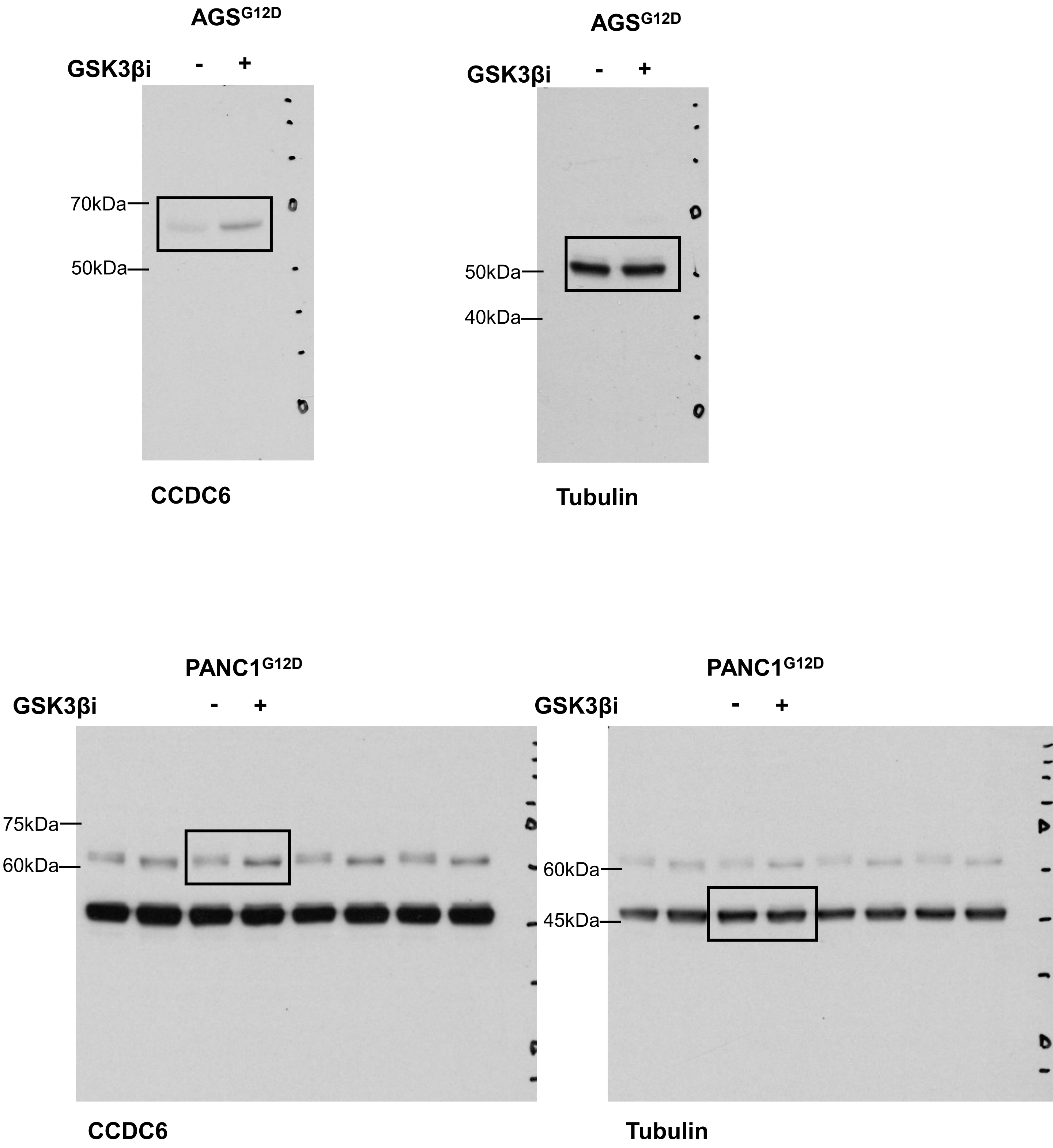
AGS^G12D
GSK3βi - +
70kDa
50kDa
CCDC6
AGS^G12D
GSK3βi - +
50kDa
40kDa
Tubulin
PANC1^G12D
GSK3βi - +
75kDa
60kDa
CCDC6
PANC1^G12D
GSK3βi - +
60kDa
45kDa
Tubulin

Original Western Blot Relative to Figure 1F

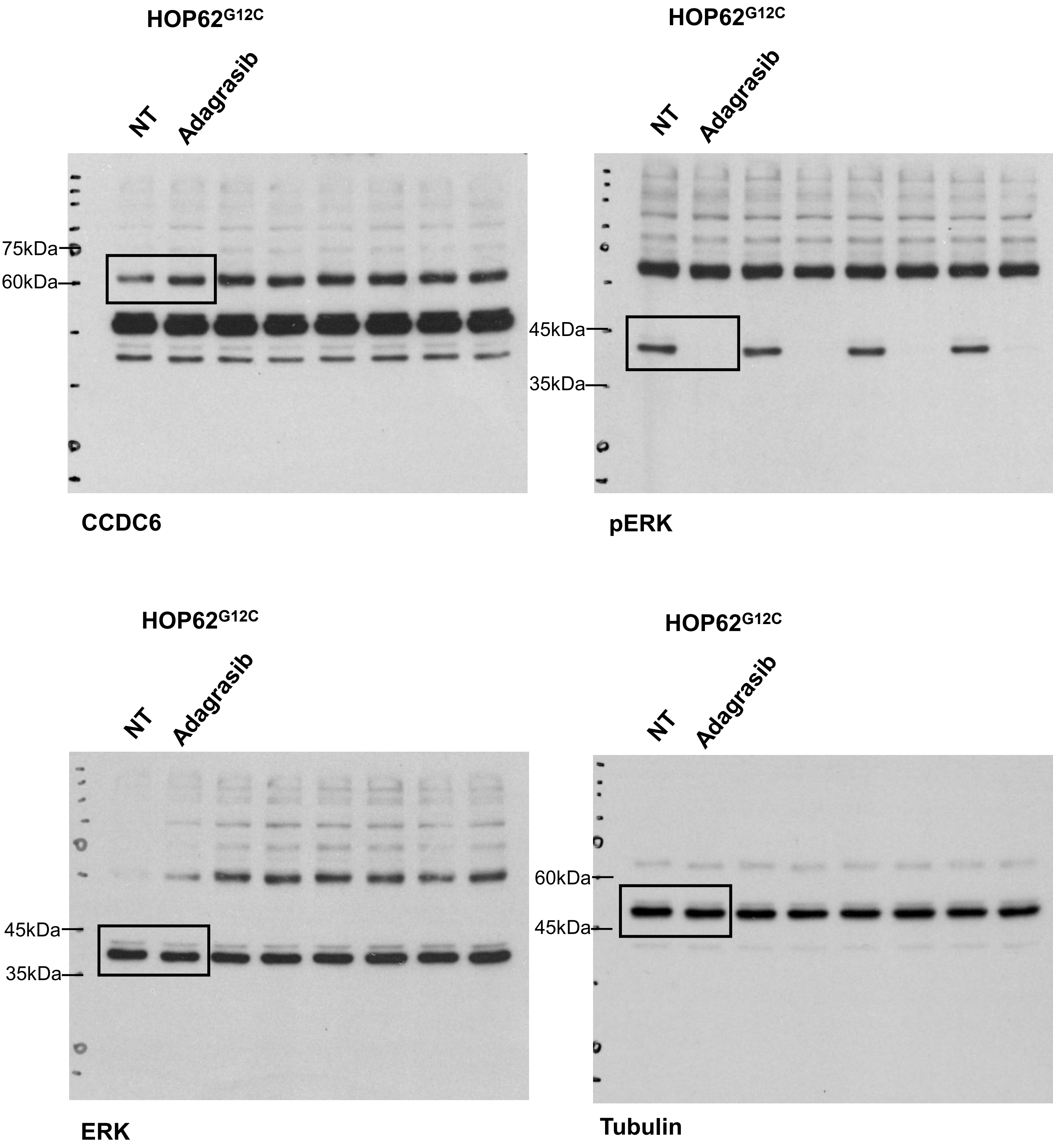

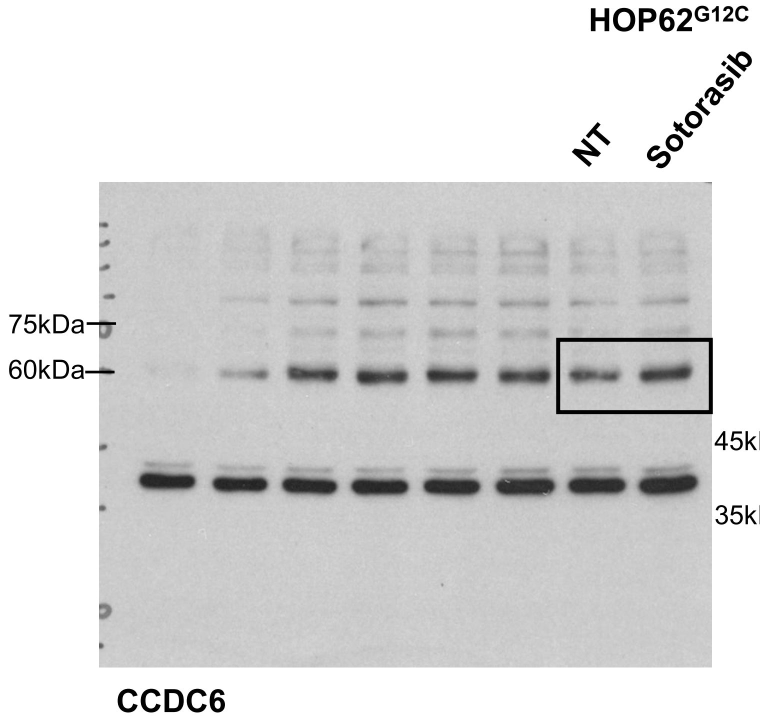
HOP62^{G12C}
NT
Sotorasib
75kDa
60kDa
CCDC6

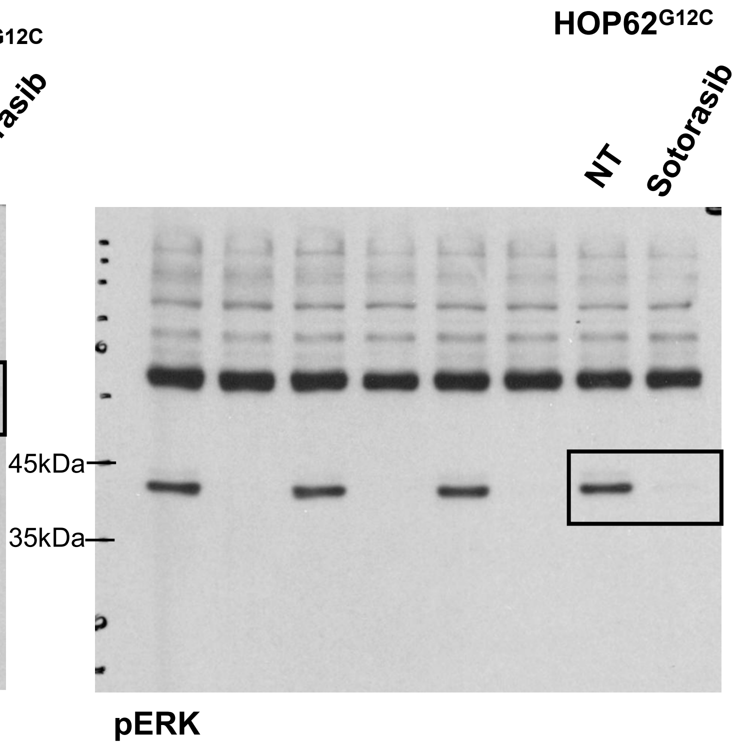
HOP62^{G12C}
NT
Sotorasib
45kDa
35kDa
pERK

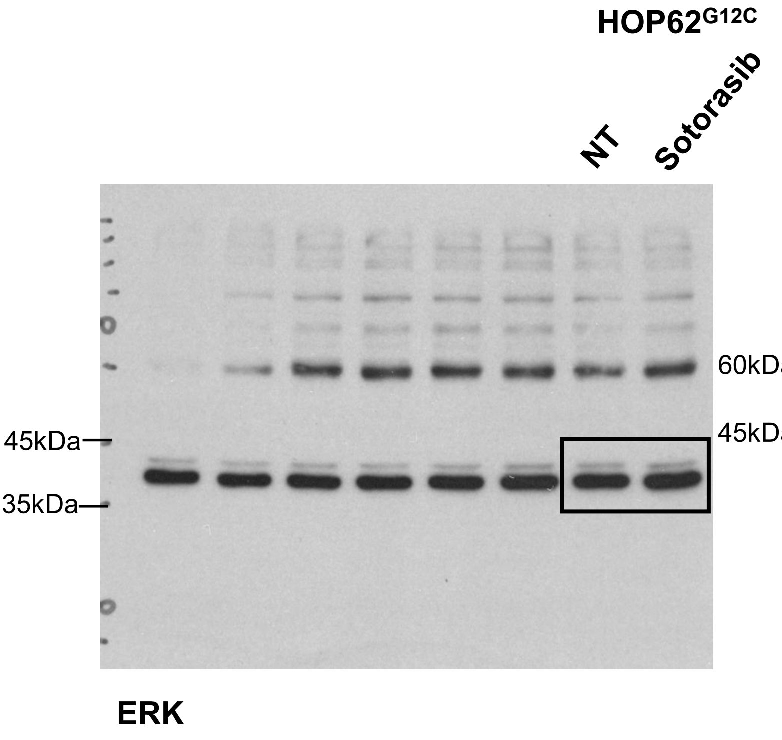
HOP62^{G12C}
NT
Sotorasib
45kDa
35kDa
ERK

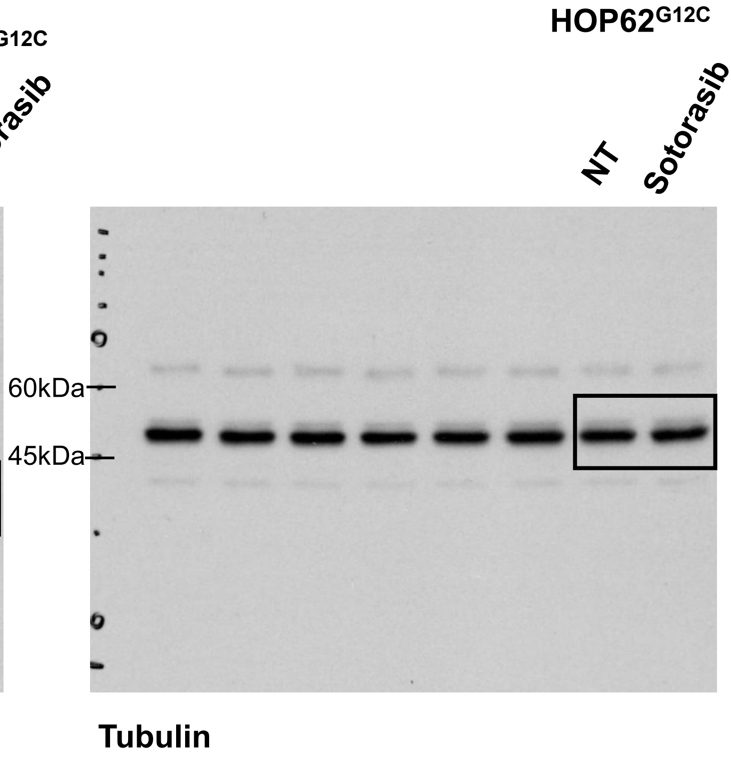
HOP62^{G12C}
NT
Sotorasib
60kDa
45kDa
Tubulin

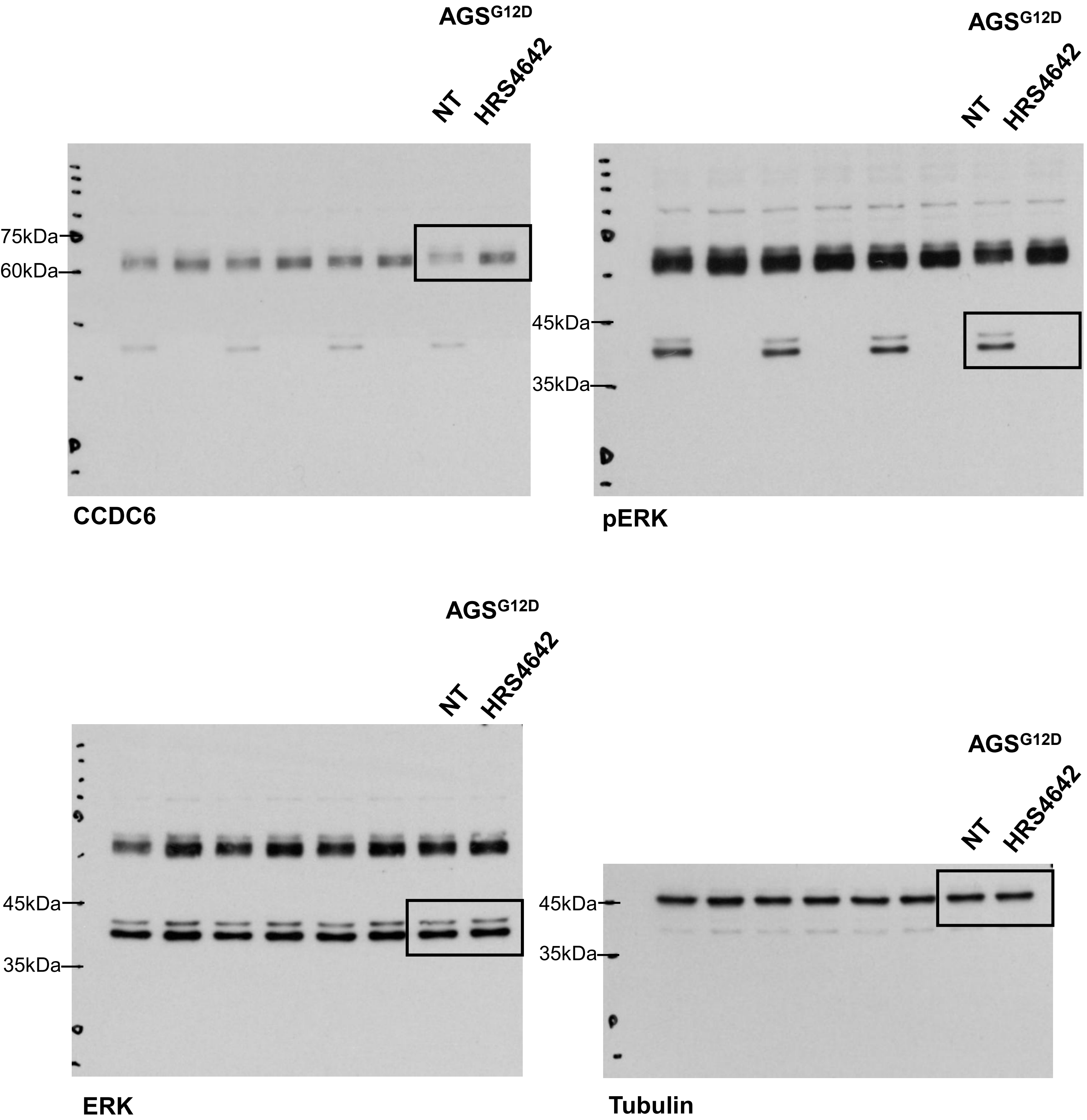

AGS^G12D
NT
HRS4642
75kDa
60kDa
CCDC6
AGS^G12D
NT
HRS4642
45kDa
35kDa
pERK
AGS^G12D
NT
HRS4642
45kDa
35kDa
ERK
AGS^G12D
NT
HRS4642
45kDa
35kDa
Tubulin

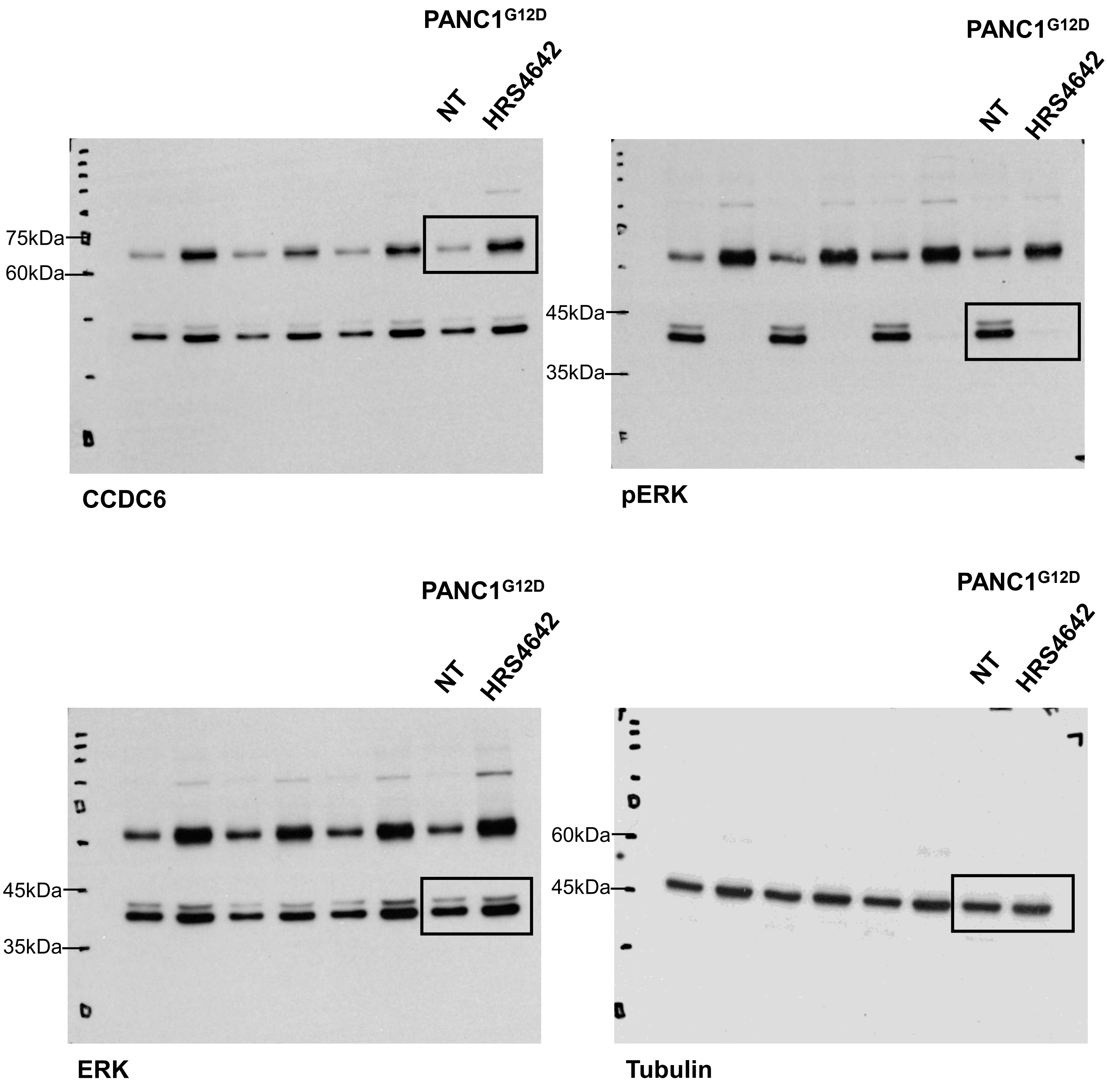
PANC1G12D
NT
HRS4642
75kDa
60kDa
CCDC6
PANC1G12D
NT
HRS4642
45kDa
35kDa
pERK
PANC1G12D
NT
HRS4642
45kDa
35kDa
ERK
PANC1G12D
NT
HRS4642
60kDa
45kDa
Tubulin

Original Western Blot Relative to Figure 1G

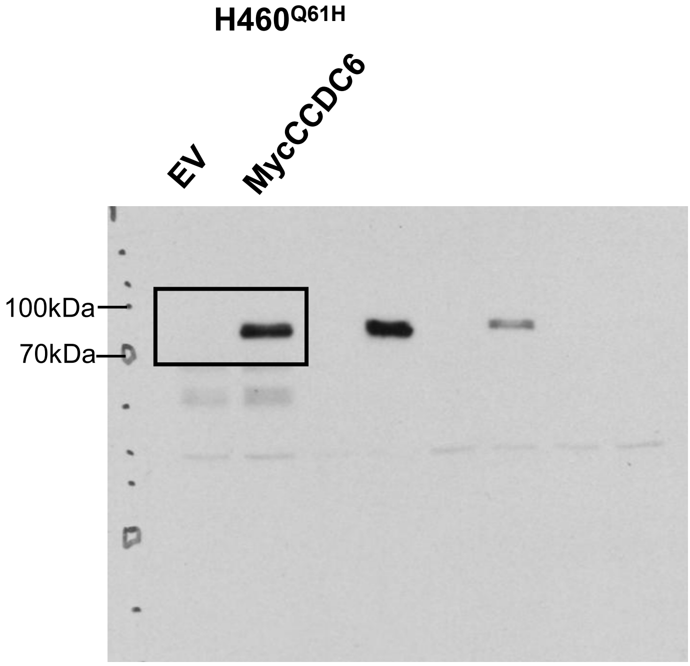

Myc

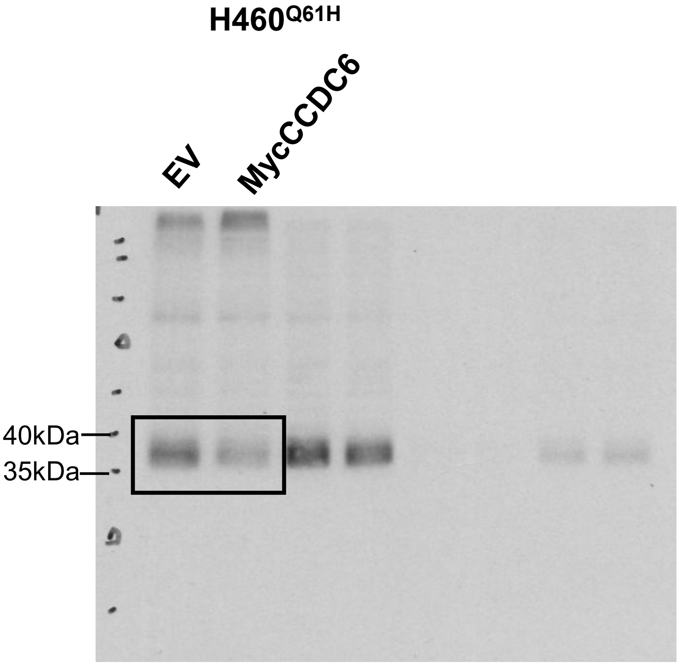

xCT

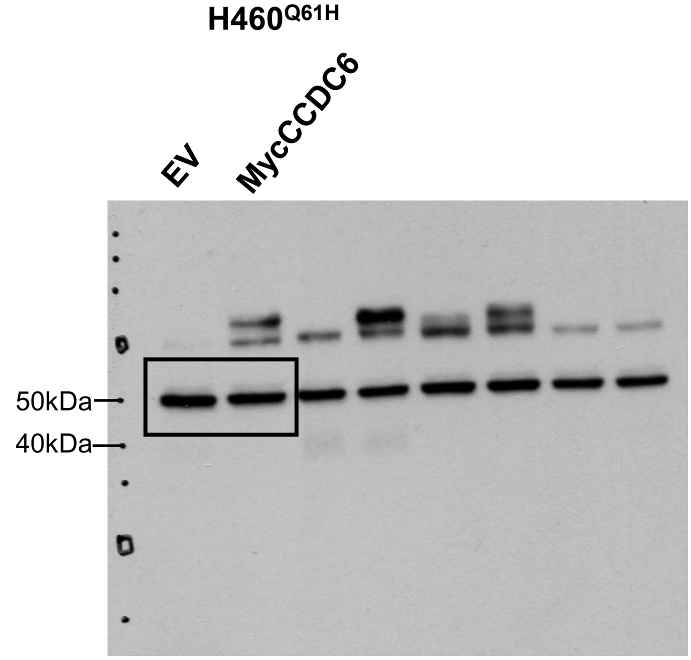

Tubulin

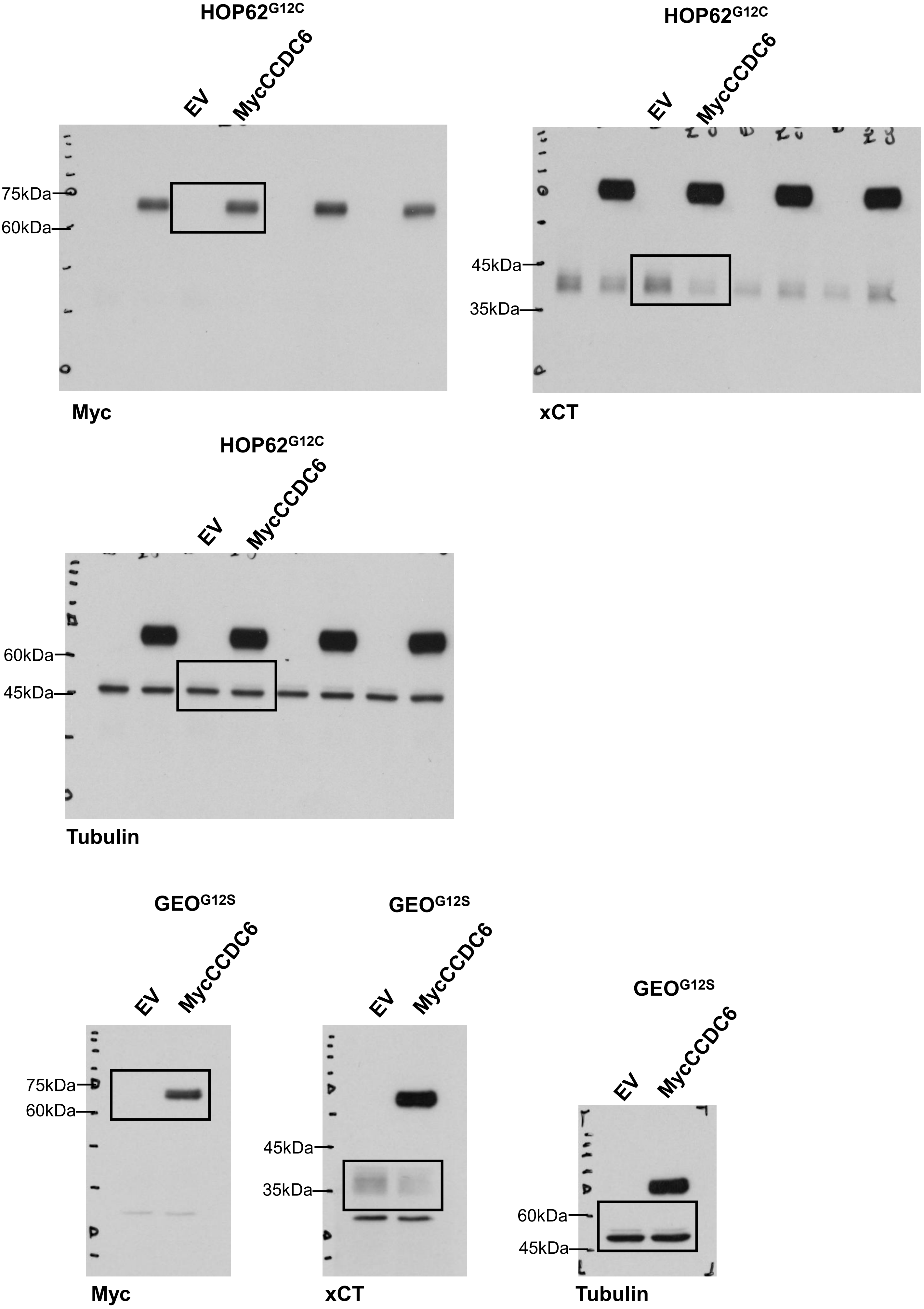

HOP62G12C
EV
MycCCDC6
75kDa
60kDa
Myc
HOP62G12C
EV
MycCCDC6
45kDa
35kDa
xCT
HOP62G12C
EV
MycCCDC6
60kDa
45kDa
Tubulin
GEOG12S
EV
MycCCDC6
75kDa
60kDa
Myc
GEOG12S
EV
MycCCDC6
45kDa
35kDa
xCT
GEOG12S
EV
MycCCDC6
60kDa
45kDa
Tubulin

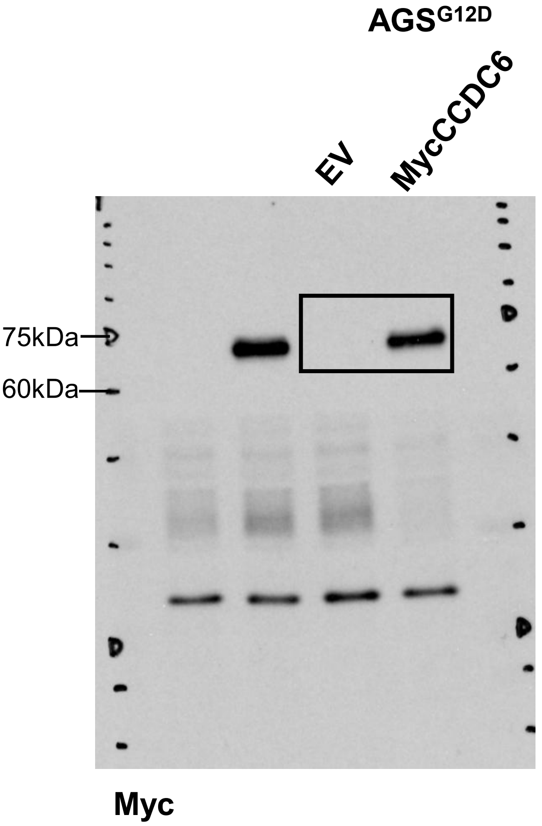

AGS^G12D
EV
MycCCDC6
75kDa
60kDa
Myc

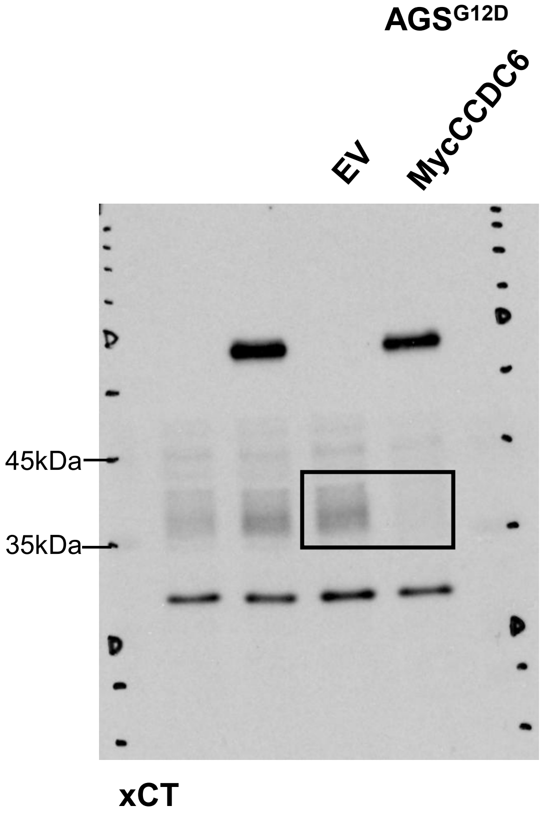

AGS^G12D
EV
MycCCDC6
45kDa
35kDa
xCT

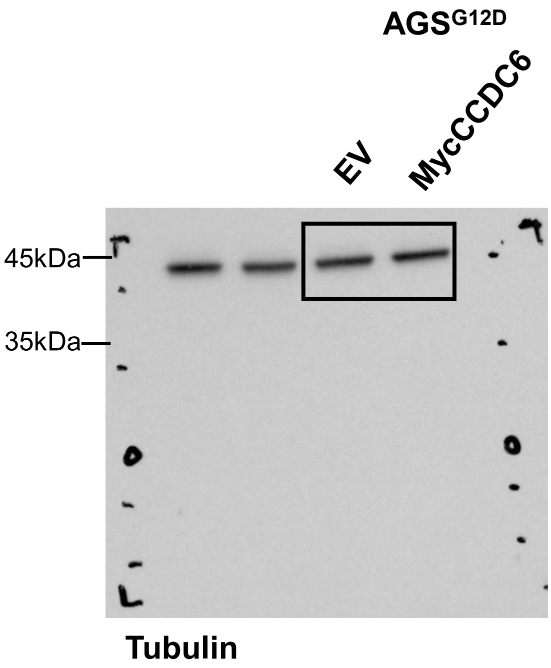

AGS^G12D
EV
MycCCDC6
45kDa
35kDa
Tubulin

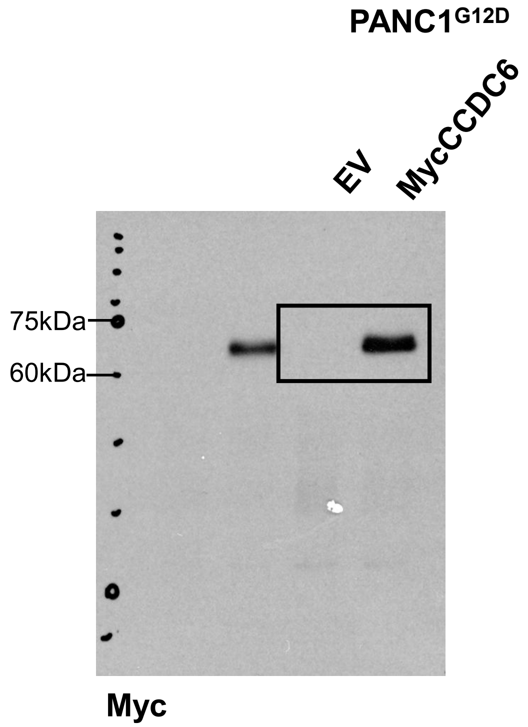
PANC1G12D
EV
MycCCDC6
75kDa
60kDa
Myc

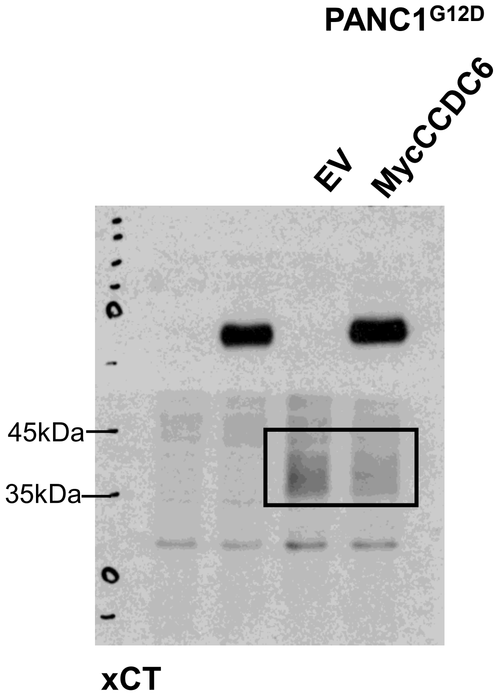
PANC1G12D
EV
MycCCDC6
45kDa
35kDa
xCT

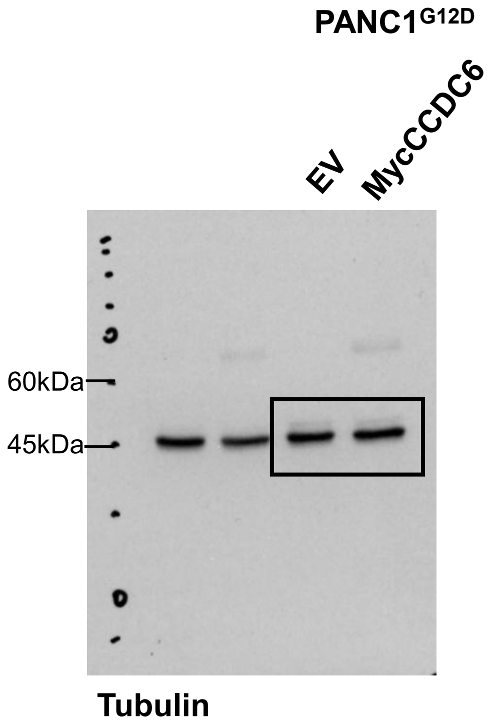
PANC1G12D
EV
MycCCDC6
60kDa
45kDa
Tubulin

Original Western Blot Relative to Figure 2A

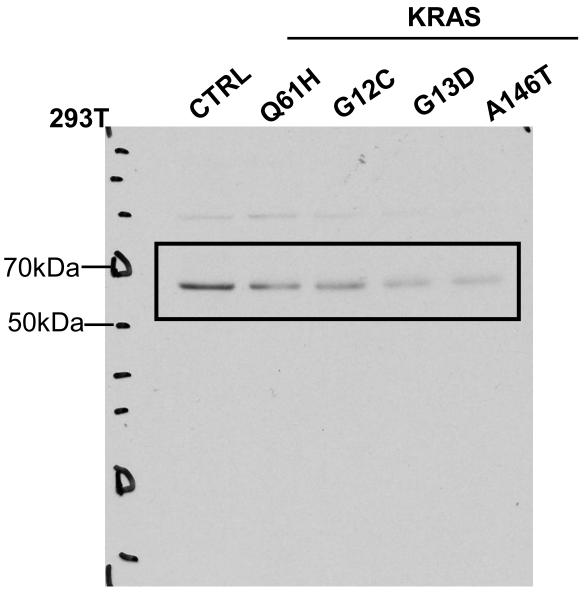

CCDC6

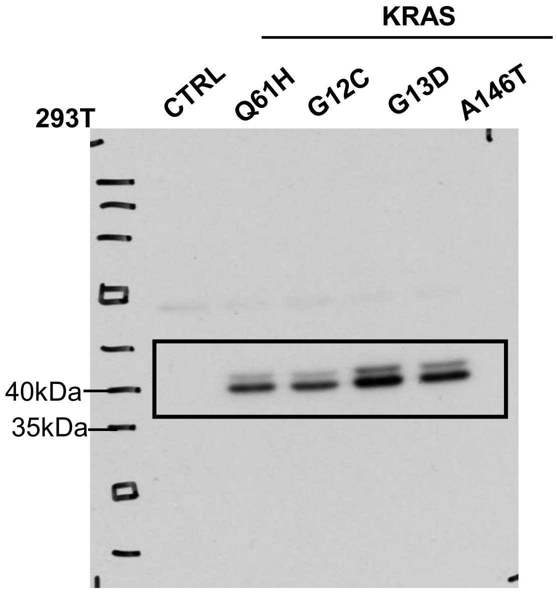

pERK

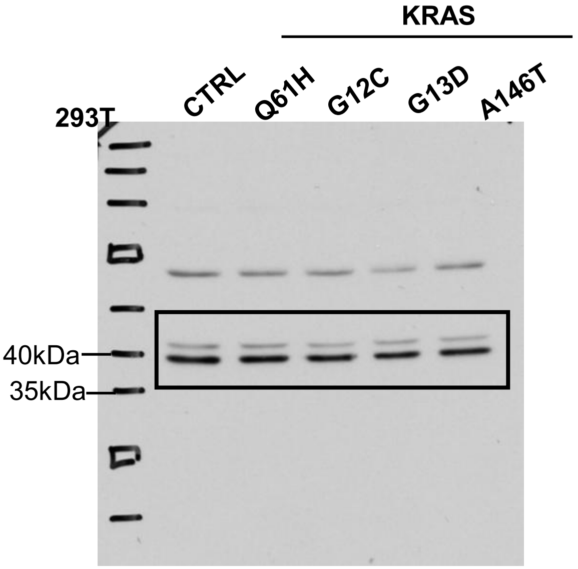

ERK

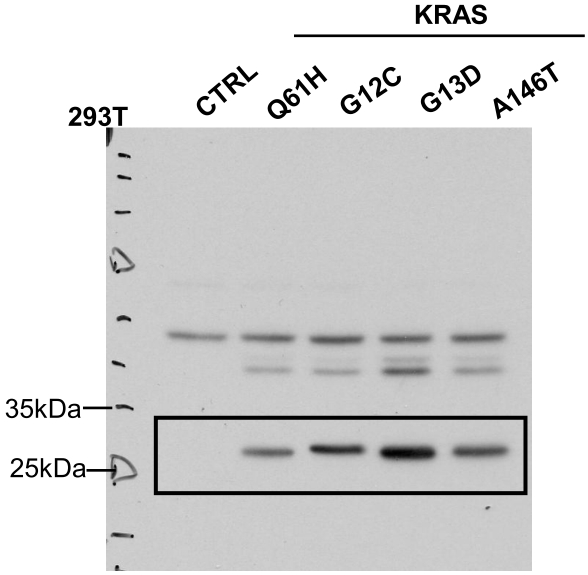

Flag

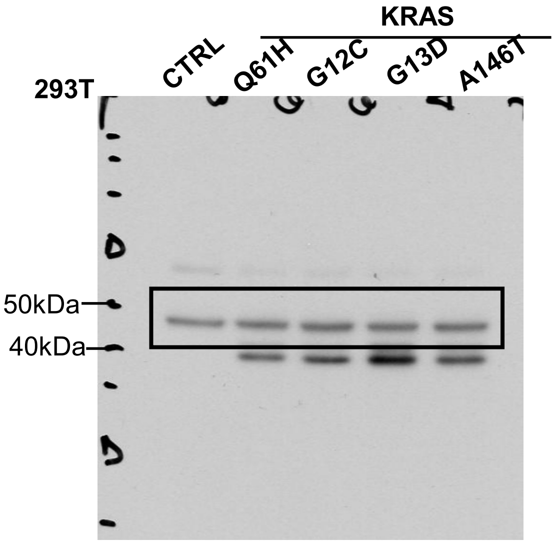

Tubulin

Original Western Blot Relative to Figure 2C

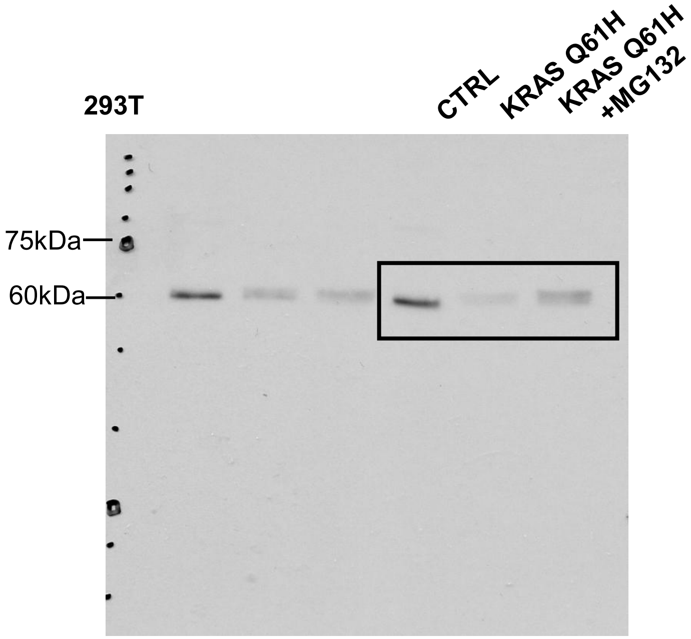

CCDC6

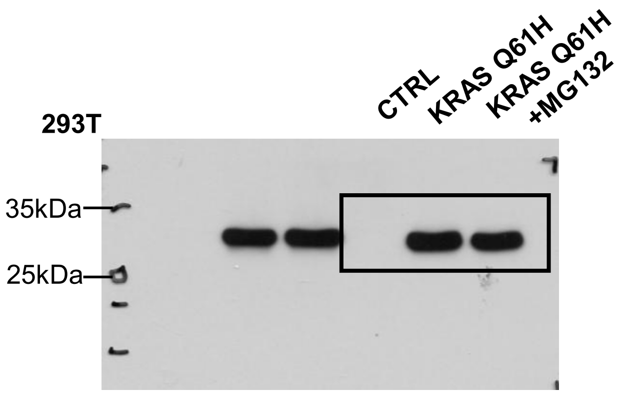

Flag

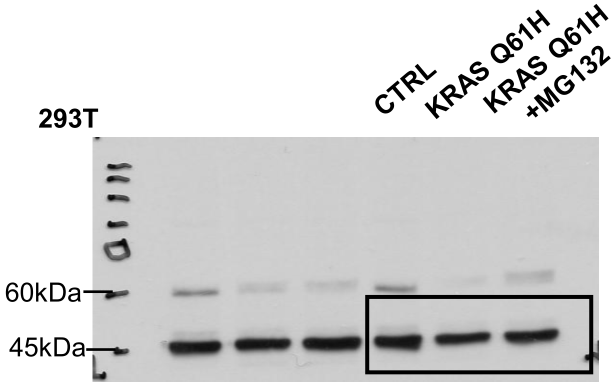

Tubulin

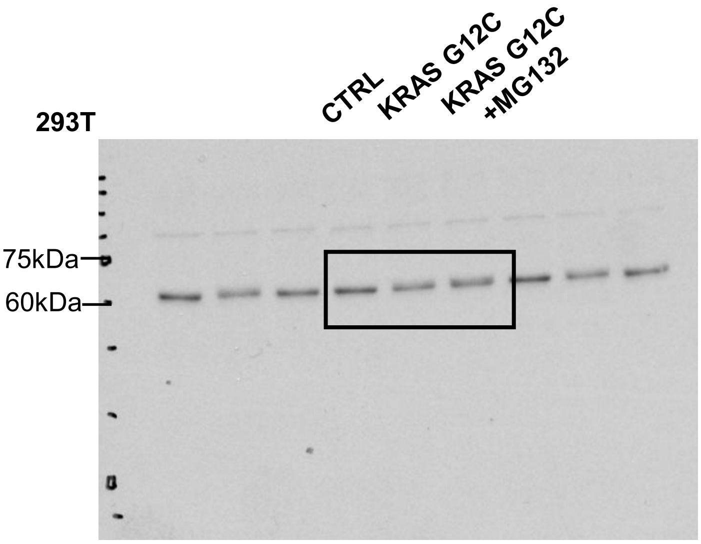

CCDC6

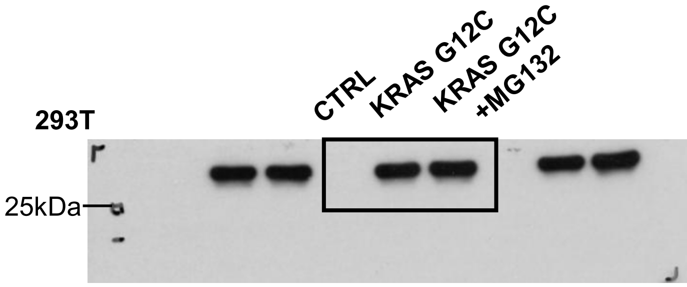

Flag

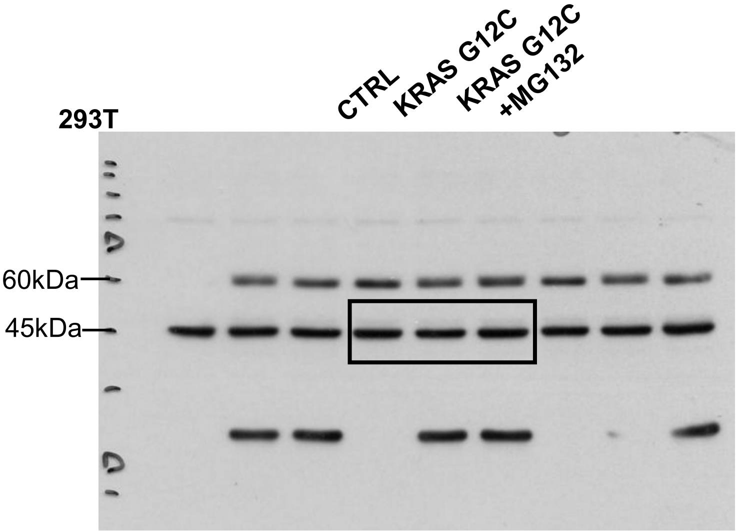

Tubulin

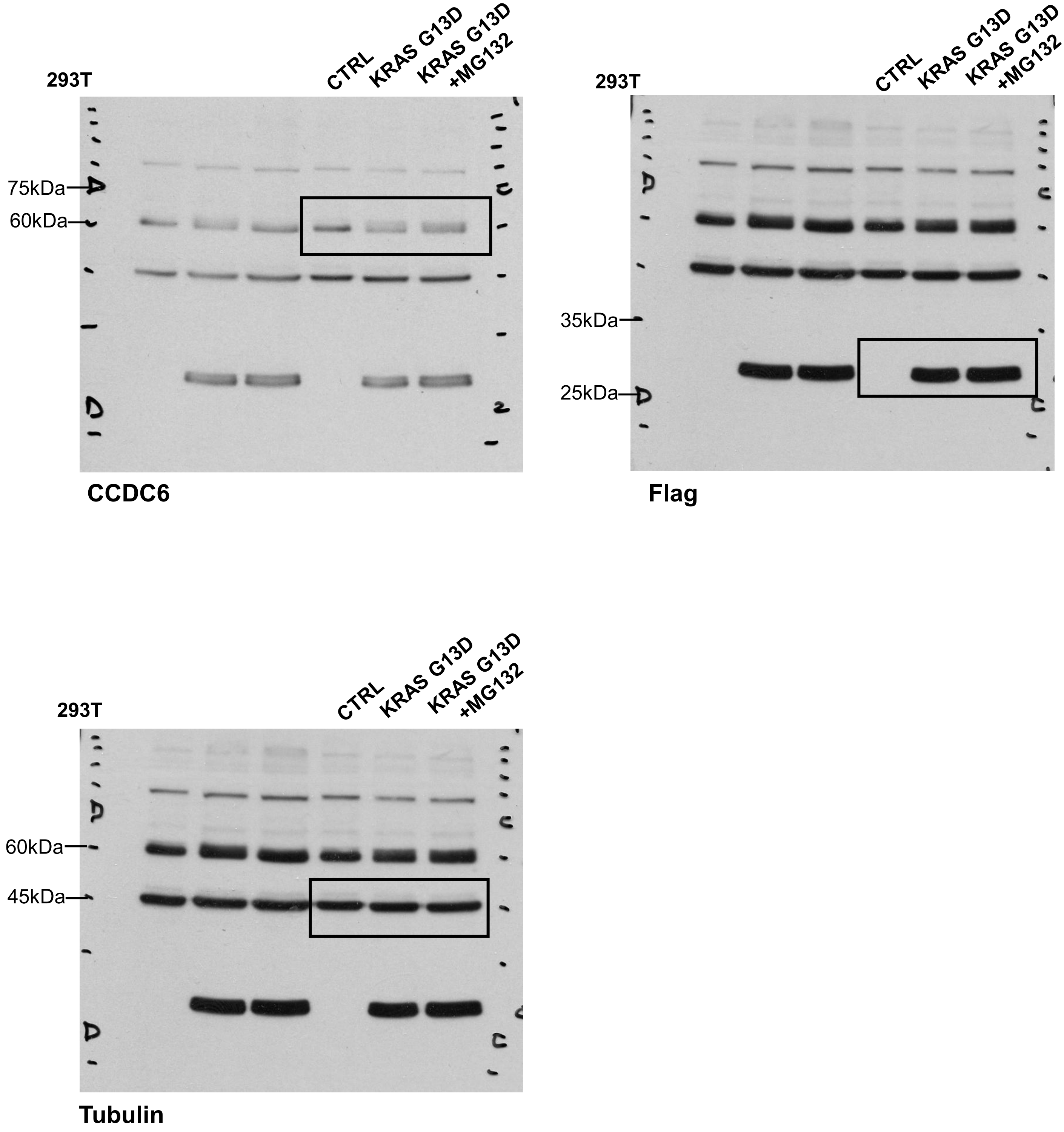
293T
CTRL
KRAS G13D
KRAS G13D +MG132
75kDa
60kDa
CCDC6
293T
CTRL
KRAS G13D
KRAS G13D +MG132
35kDa
25kDa
Flag
293T
CTRL
KRAS G13D
KRAS G13D +MG132
60kDa
45kDa
Tubulin

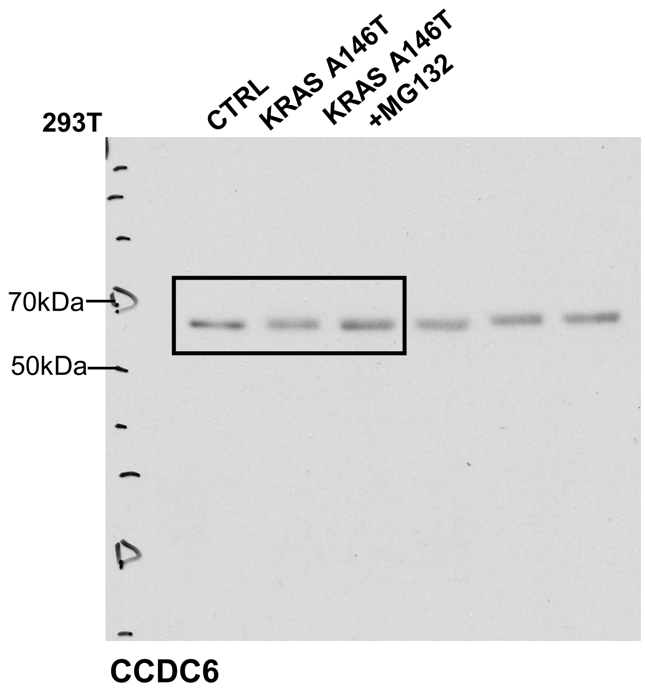
293T
CTRL
KRAS A146T
KRAS A146T +MG132
70kDa
50kDa
CCDC6

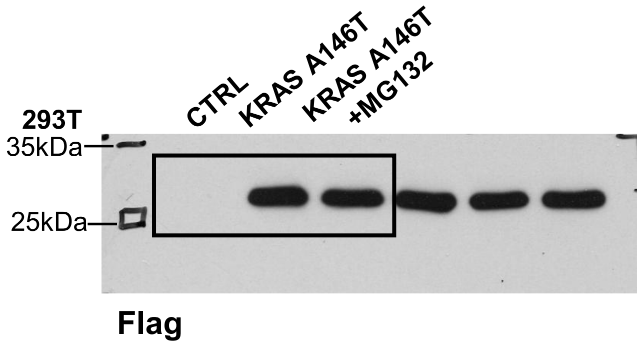
293T
CTRL
KRAS A146T
KRAS A146T +MG132
35kDa
25kDa
Flag

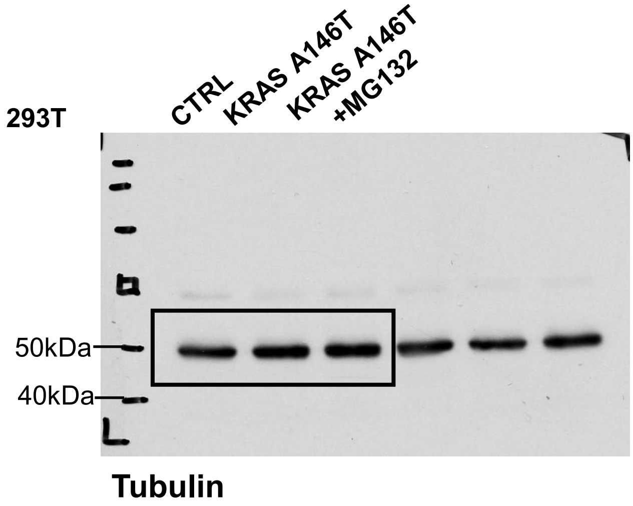
293T
CTRL
KRAS A146T
KRAS A146T +MG132
50kDa
40kDa
Tubulin

Original Western Blot Relative to Figure 2D

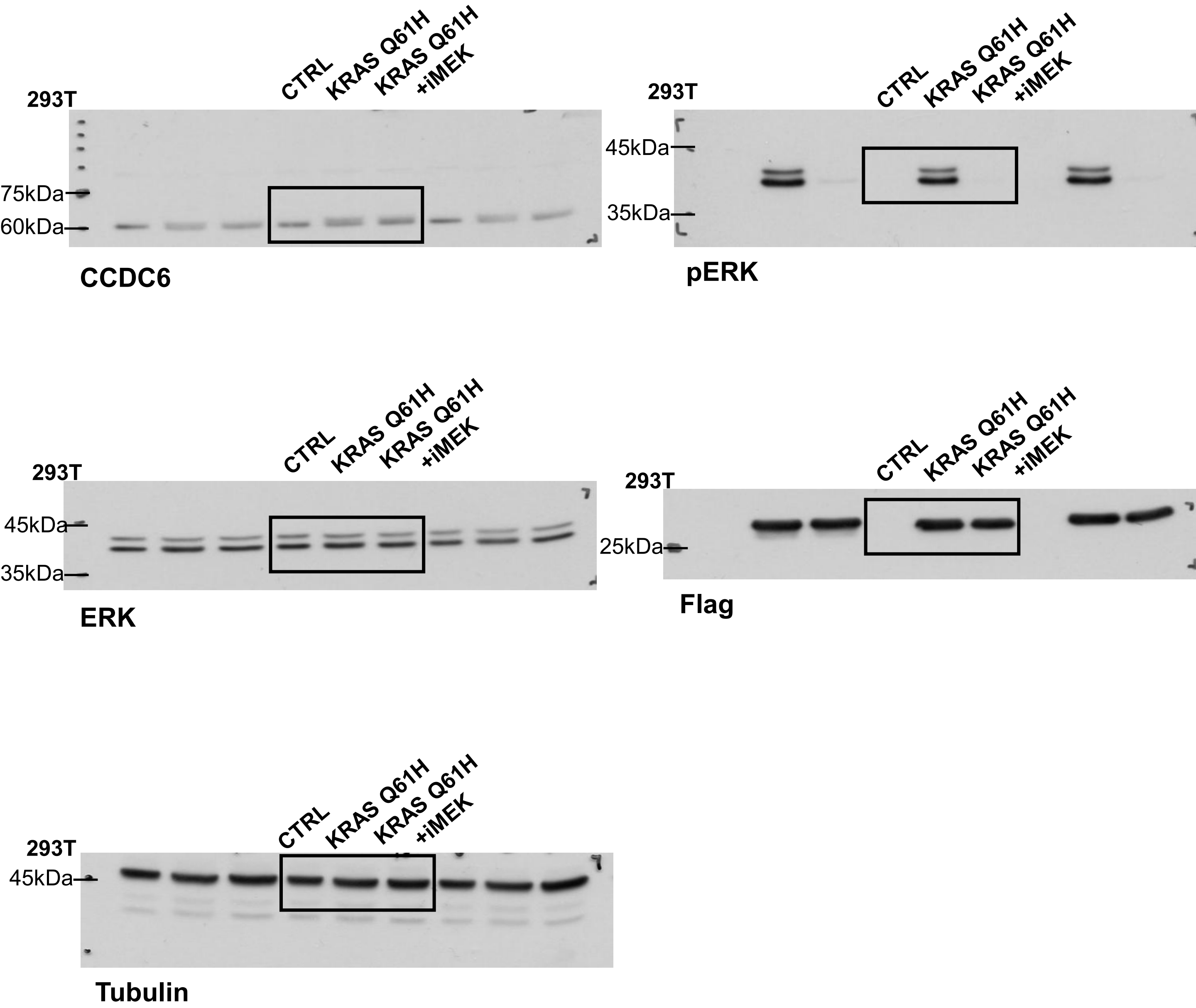

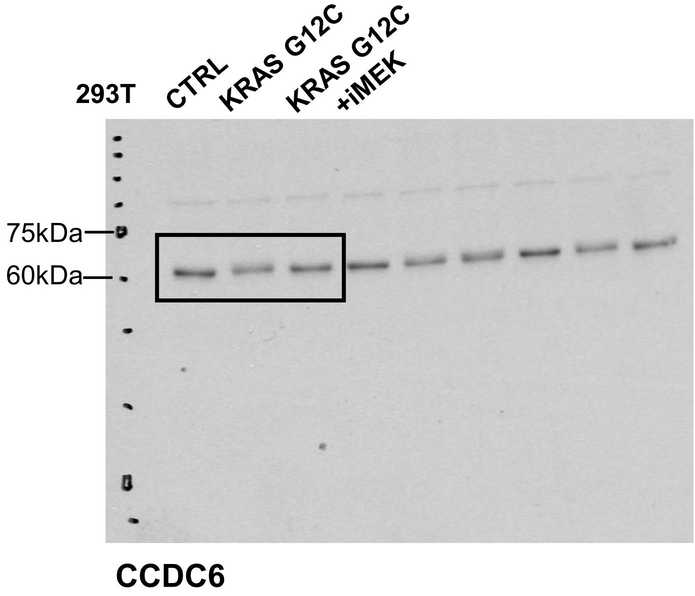
293T
CTRL
KRAS G12C
KRAS G12C
+iMEK
75kDa
60kDa
CCDC6

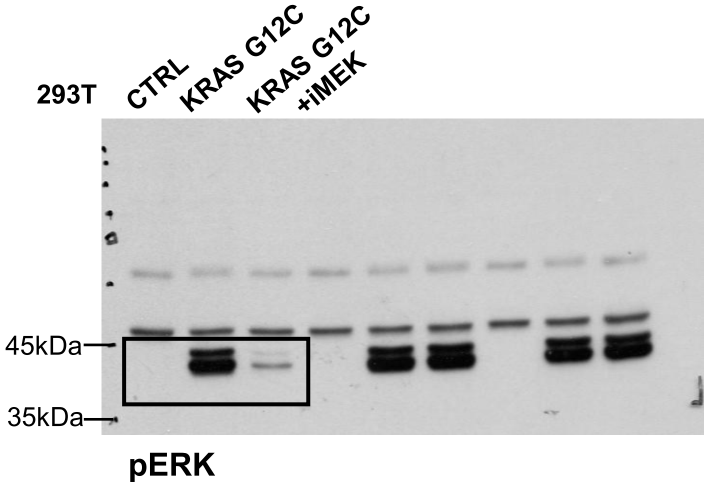
293T
CTRL
KRAS G12C
KRAS G12C
+iMEK
45kDa
35kDa
pERK

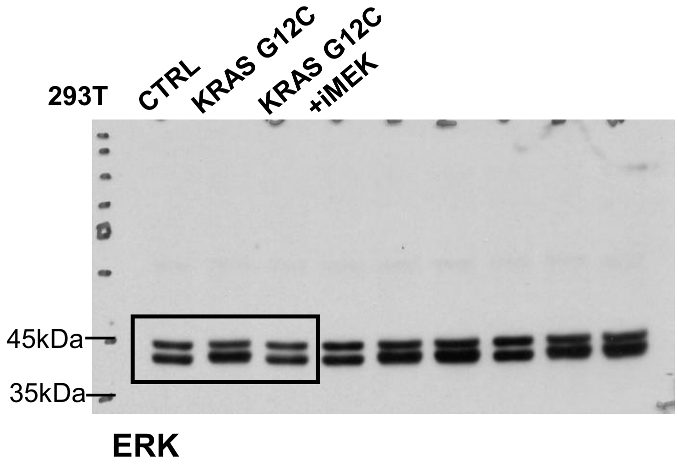
293T
CTRL
KRAS G12C
KRAS G12C
+iMEK
45kDa
35kDa
ERK

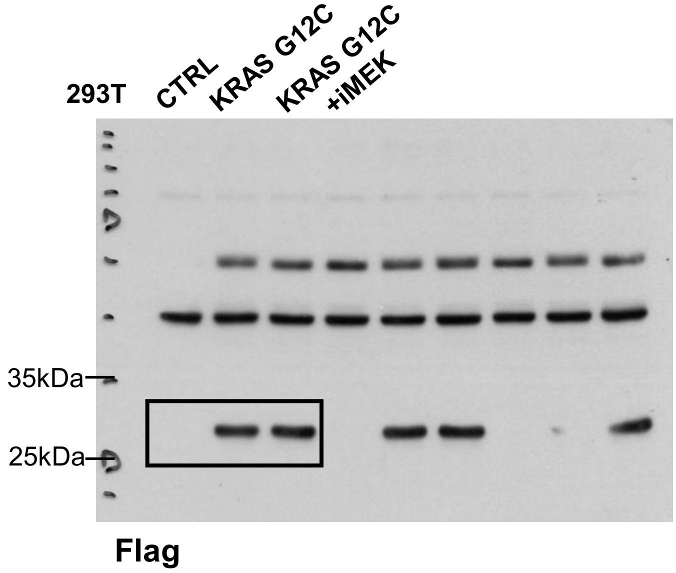
293T
CTRL
KRAS G12C
KRAS G12C
+iMEK
35kDa
25kDa
Flag

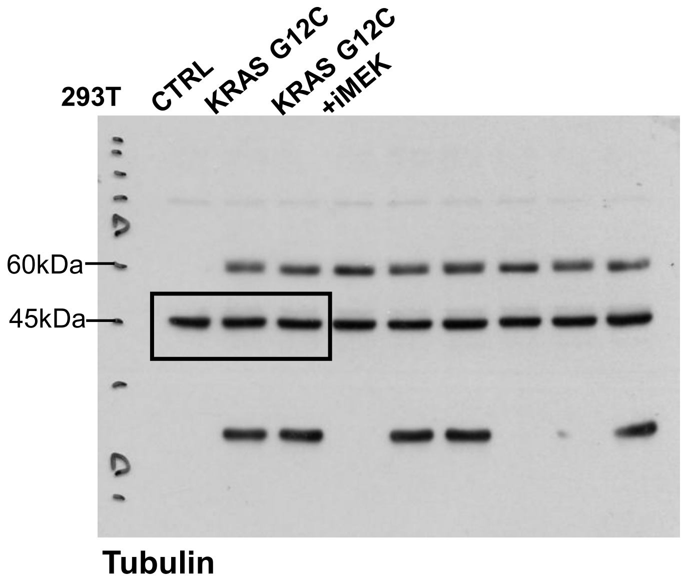
293T
CTRL
KRAS G12C
KRAS G12C
+iMEK
60kDa
45kDa
Tubulin

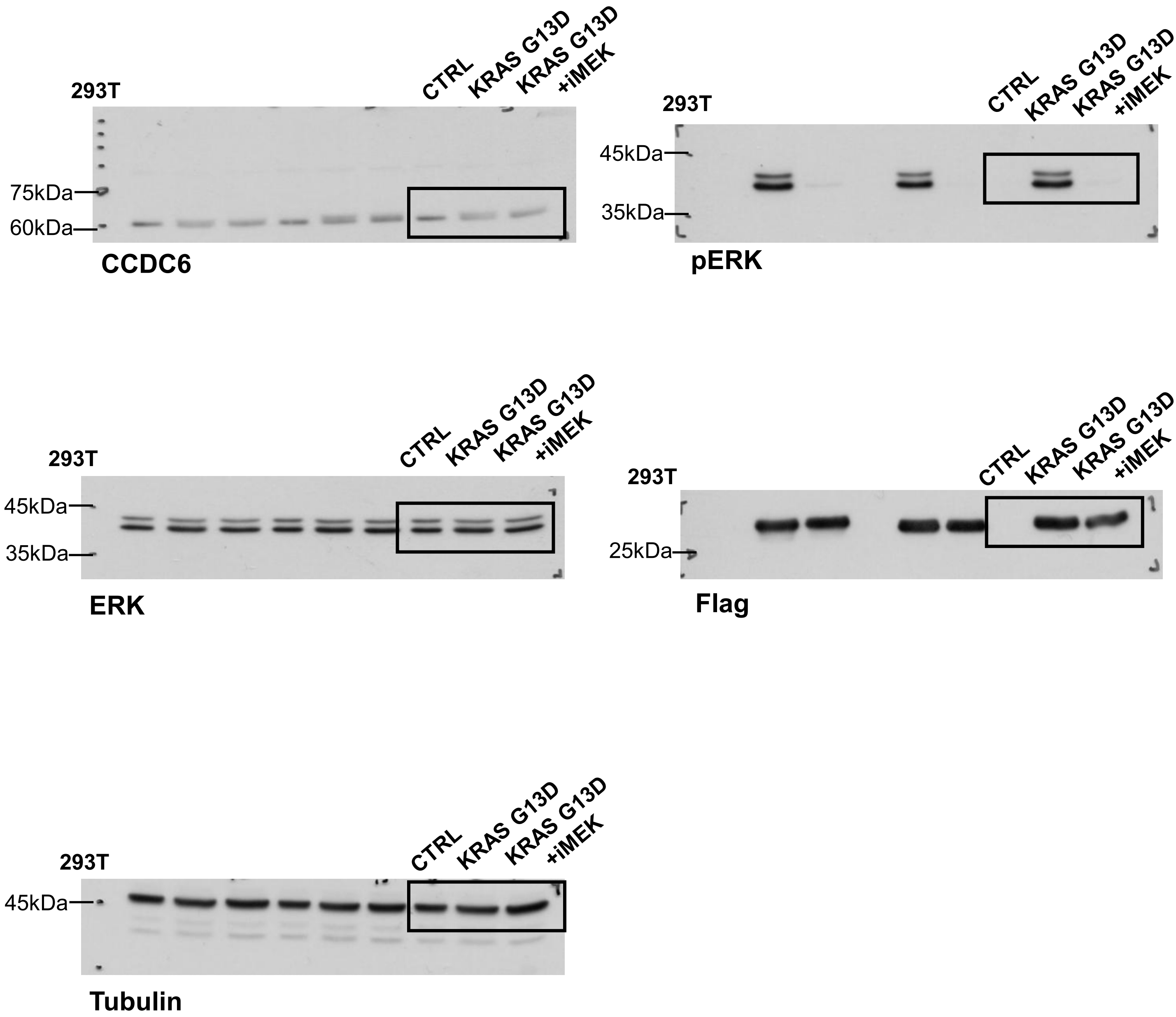

293T
CTRL
KRAS G13D
KRAS G13D +iMEK
75kDa
60kDa
CCDC6
293T
CTRL
KRAS G13D
KRAS G13D +iMEK
45kDa
35kDa
pERK
293T
CTRL
KRAS G13D
KRAS G13D +iMEK
45kDa
35kDa
ERK
293T
CTRL
KRAS G13D
KRAS G13D +iMEK
25kDa
Flag
293T
CTRL
KRAS G13D
KRAS G13D +iMEK
45kDa
Tubulin

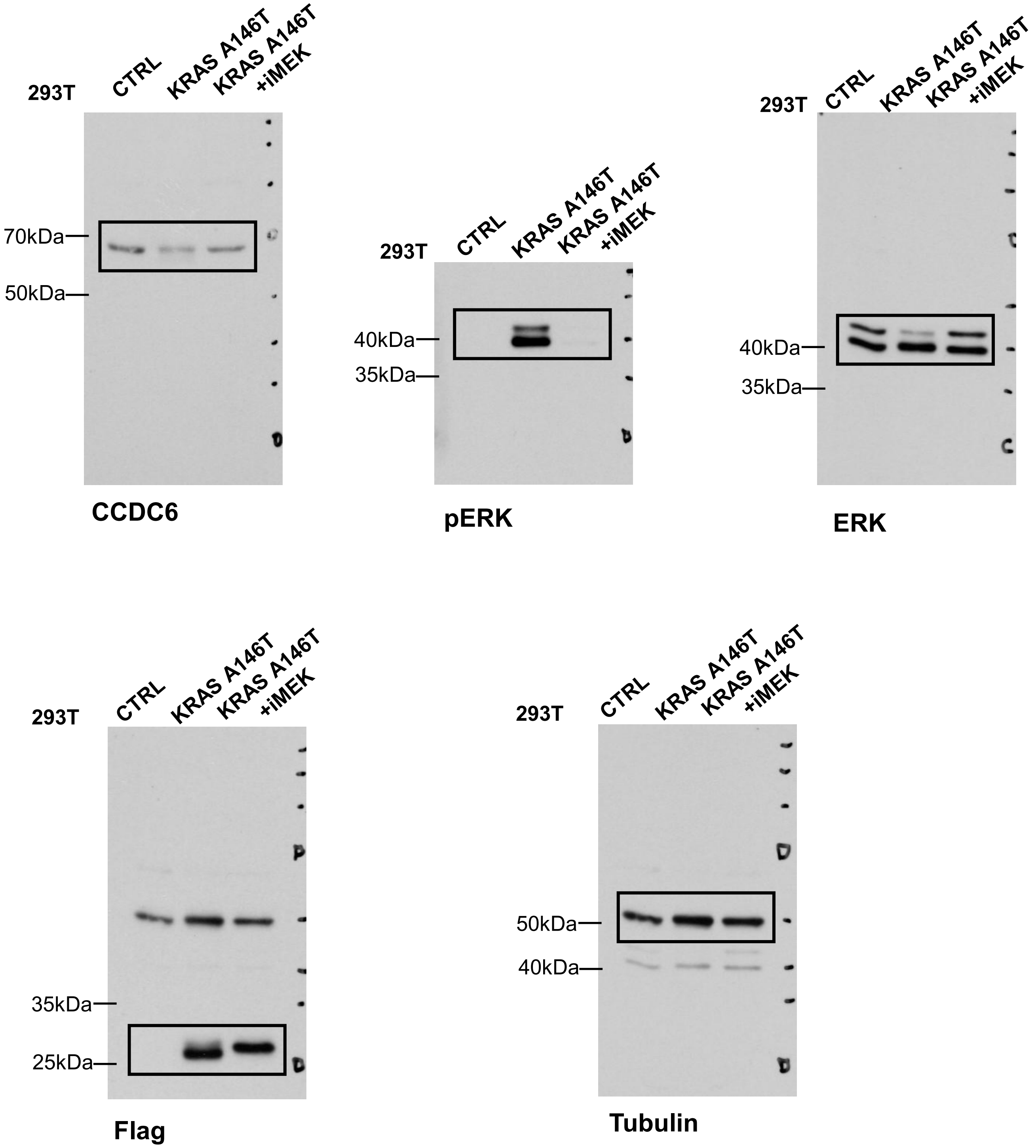
293T
CTRL
KRAS A146T
KRAS A146T +iMEK
70kDa
50kDa
CCDC6
293T
CTRL
KRAS A146T
KRAS A146T +iMEK
40kDa
35kDa
pERK
293T
CTRL
KRAS A146T
KRAS A146T +iMEK
40kDa
35kDa
ERK
293T
CTRL
KRAS A146T
KRAS A146T +iMEK
35kDa
25kDa
Flag
293T
CTRL
KRAS A146T
KRAS A146T +iMEK
50kDa
40kDa
Tubulin

Original Western Blot Relative to Figure 2E

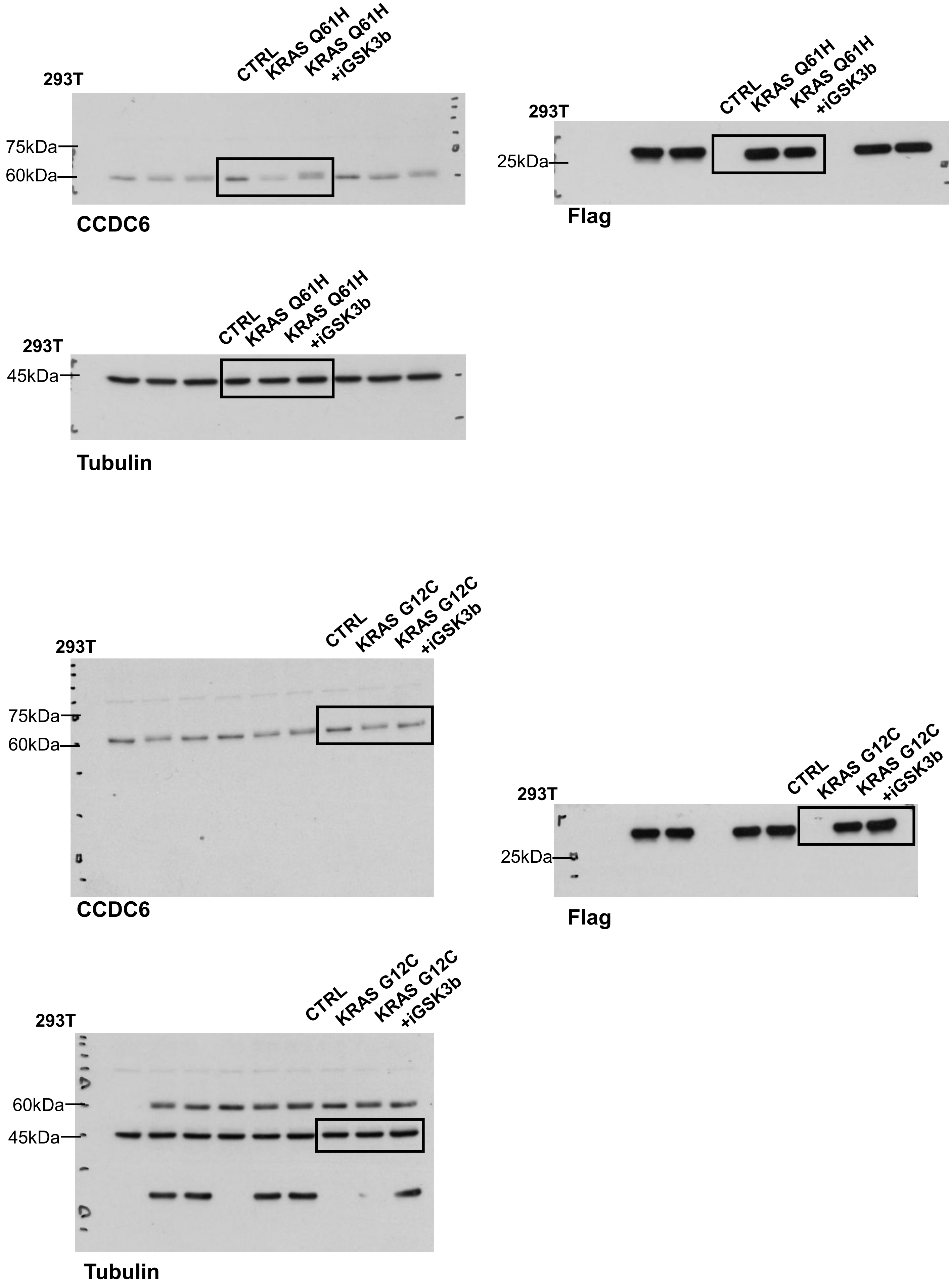

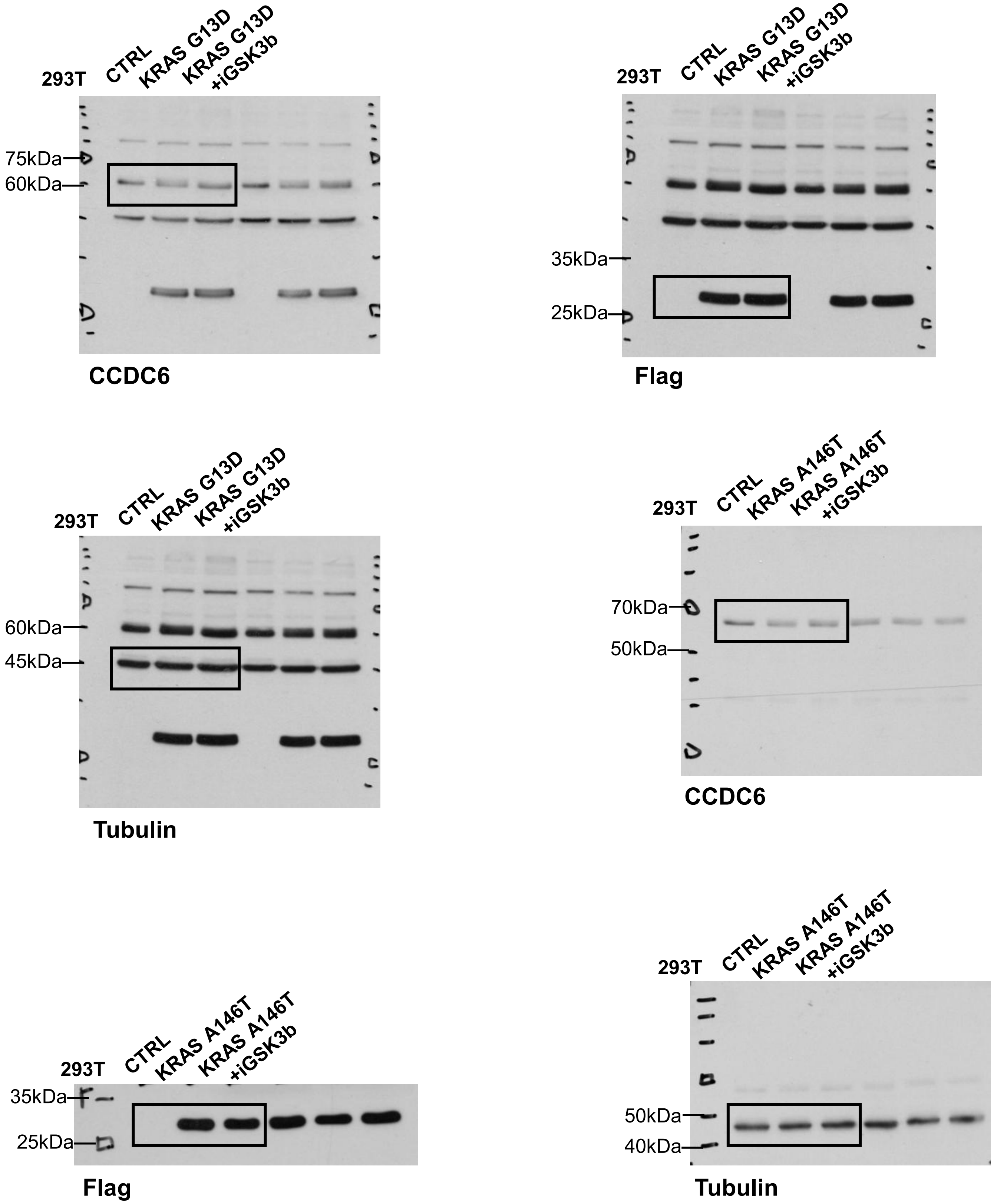

293T
CTRL
KRAS G13D
KRAS G13D +iGSK3b
75kDa
60kDa
CCDC6
293T
CTRL
KRAS G13D
KRAS G13D +iGSK3b
35kDa
25kDa
Flag
293T
CTRL
KRAS G13D
KRAS G13D +iGSK3b
60kDa
45kDa
Tubulin
293T
CTRL
KRAS A146T
KRAS A146T +iGSK3b
70kDa
50kDa
CCDC6
293T
CTRL
KRAS A146T
KRAS A146T +iGSK3b
35kDa
25kDa
Flag
293T
CTRL
KRAS A146T
KRAS A146T +iGSK3b
50kDa
40kDa
Tubulin

Original Western Blot Relative to Figure 2F

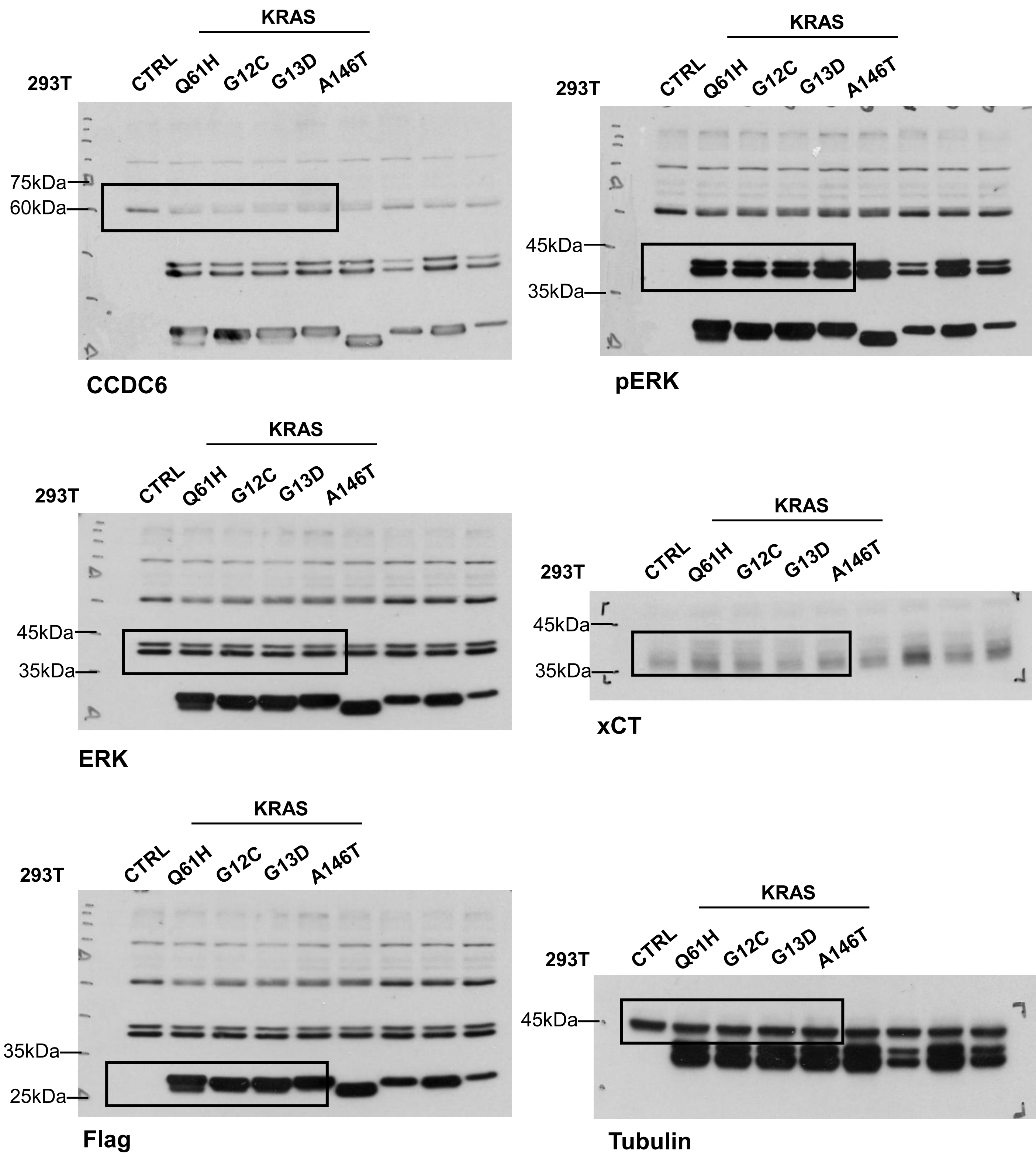

Original Western Blot Relative to Figure 2H

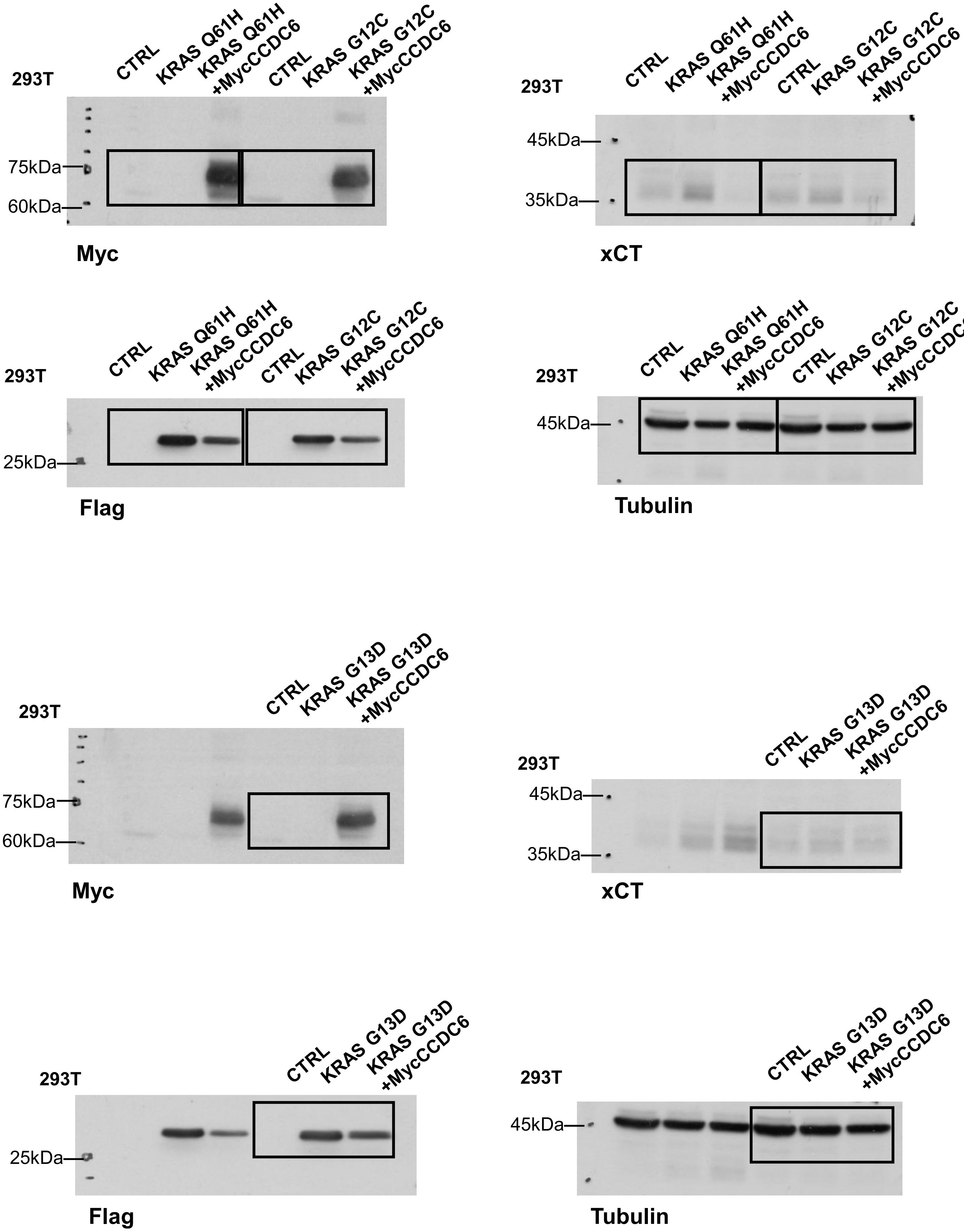

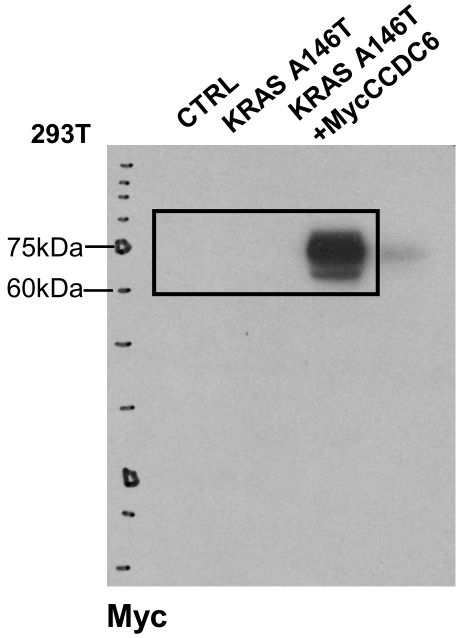
293T
CTRL
KRAS A146T
KRAS A146T +MycCCDC6
75kDa
60kDa
Myc

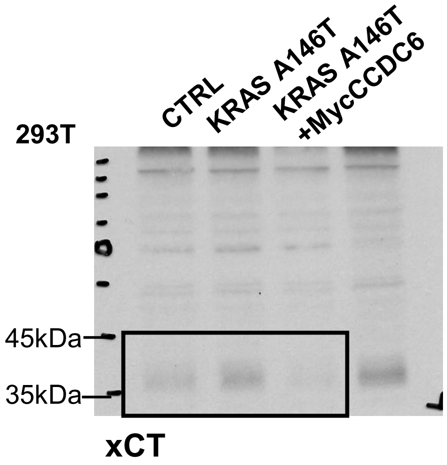
293T
CTRL
KRAS A146T
KRAS A146T +MycCCDC6
45kDa
35kDa
xCT

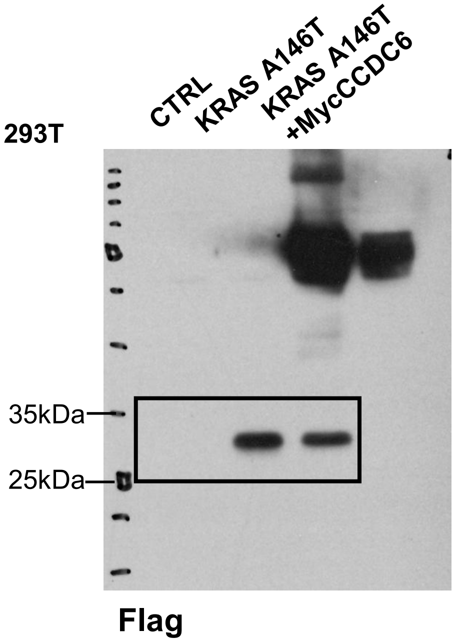
293T
CTRL
KRAS A146T
KRAS A146T +MycCCDC6
35kDa
25kDa
Flag

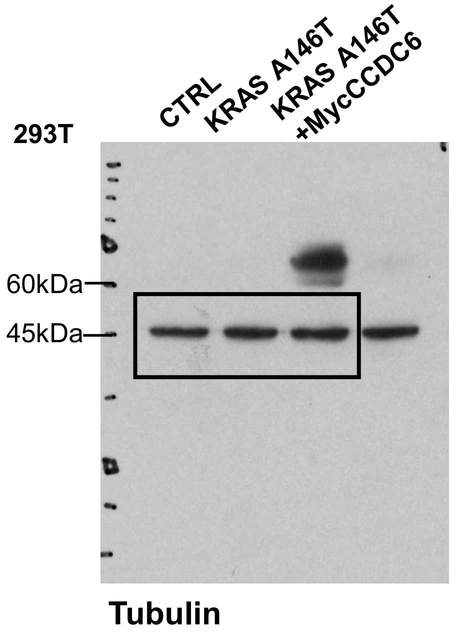
293T
CTRL
KRAS A146T
KRAS A146T +MycCCDC6
60kDa
45kDa
Tubulin

Original Western Blot Relative to Figure 3A

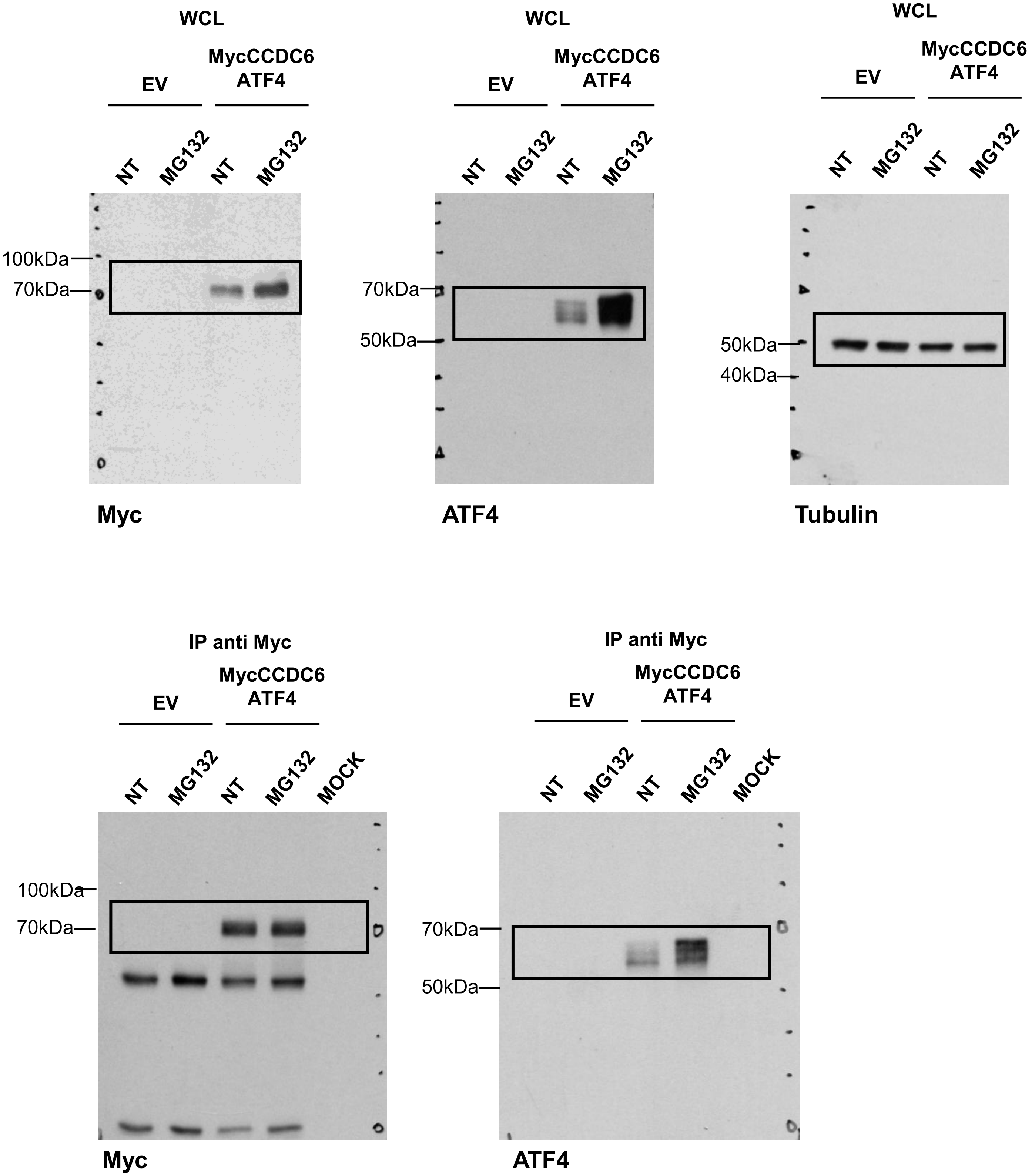

Original Western Blot Relative to Figure 3B

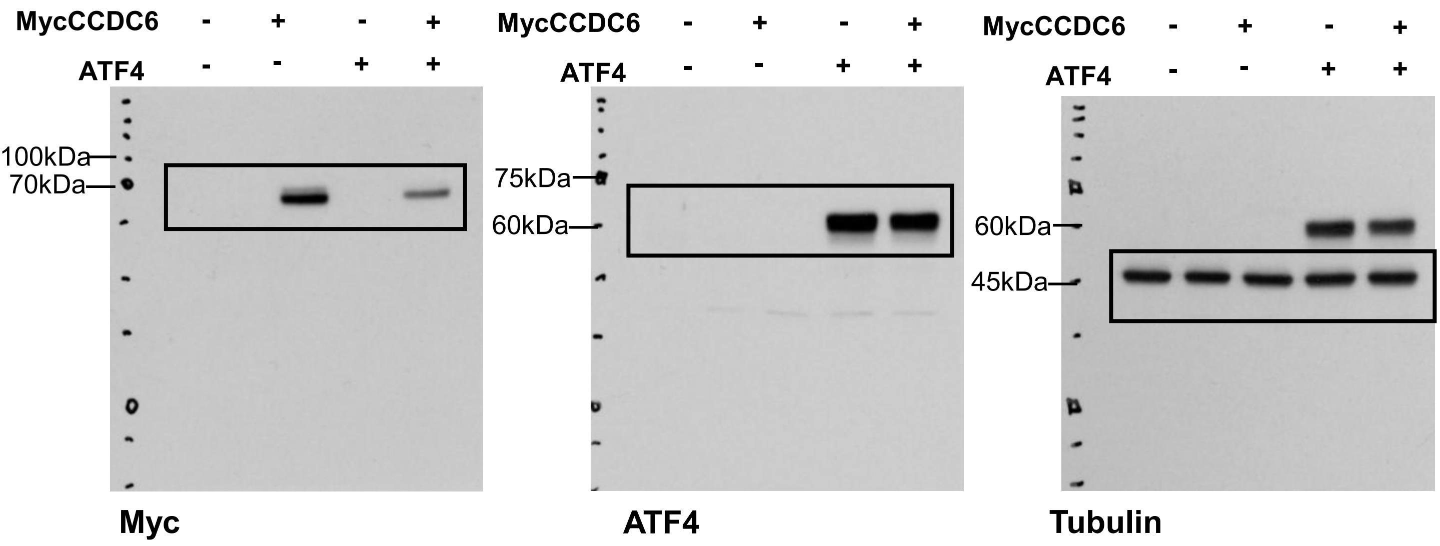

Original Western Blot Relative to Figure 3C

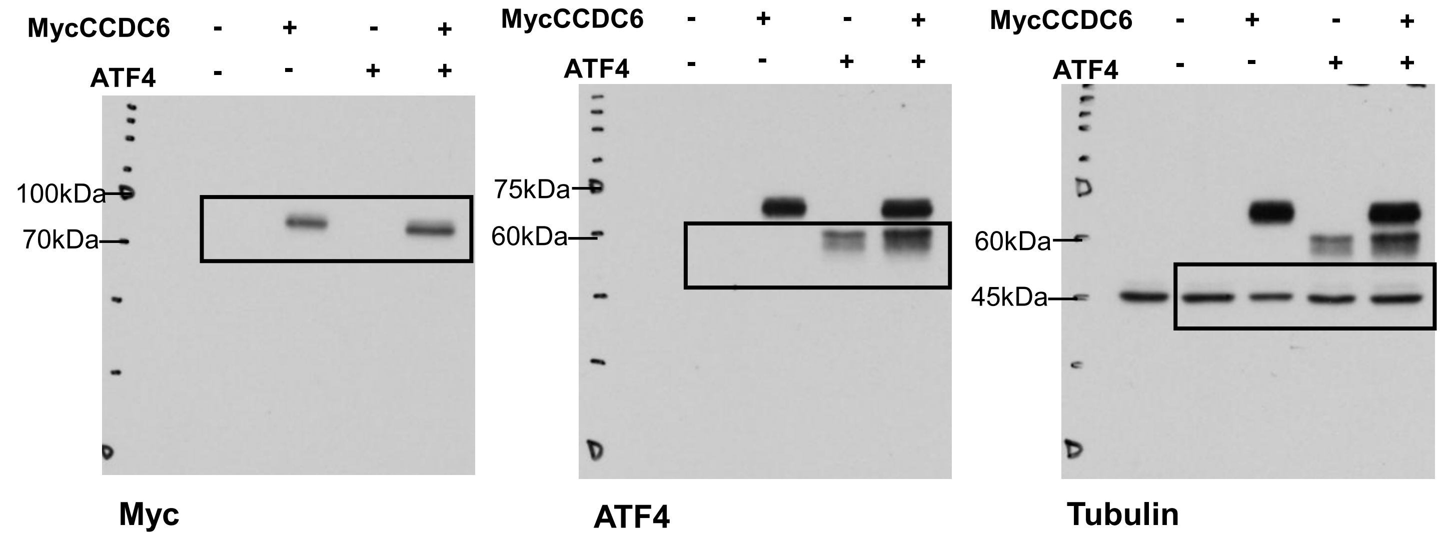

Original Western Blot Relative to Figure 5C

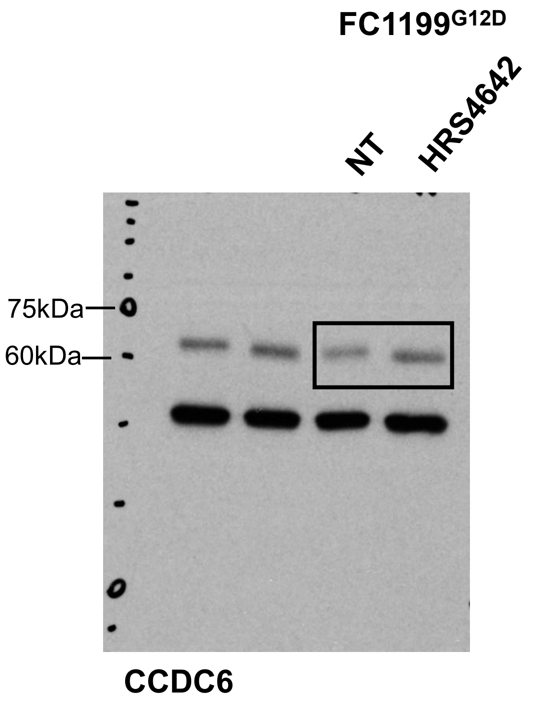

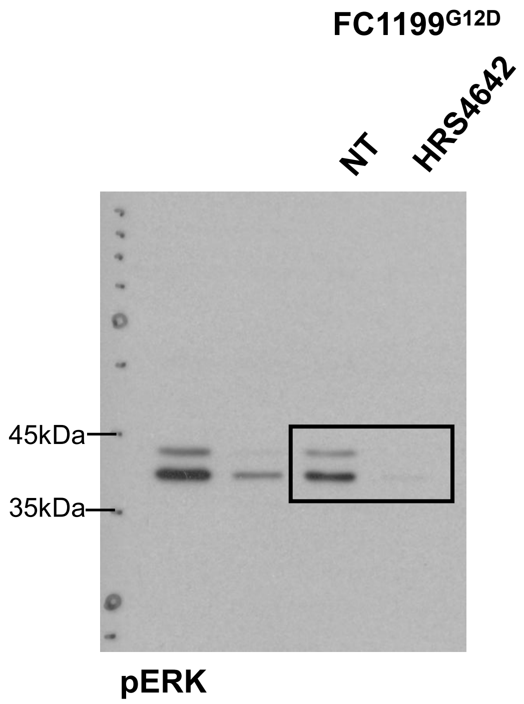

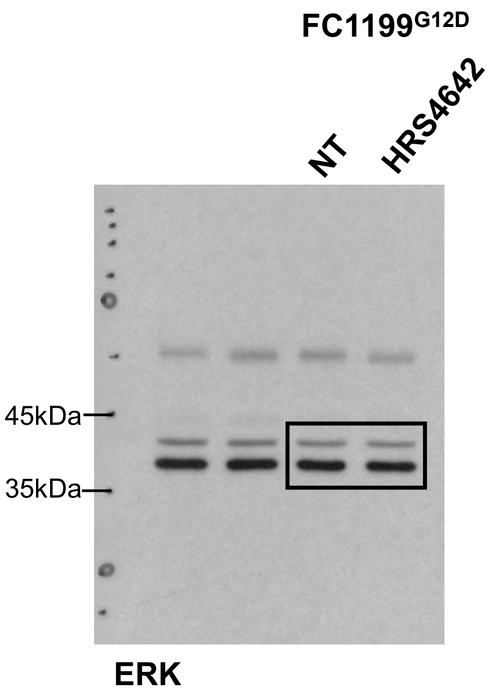

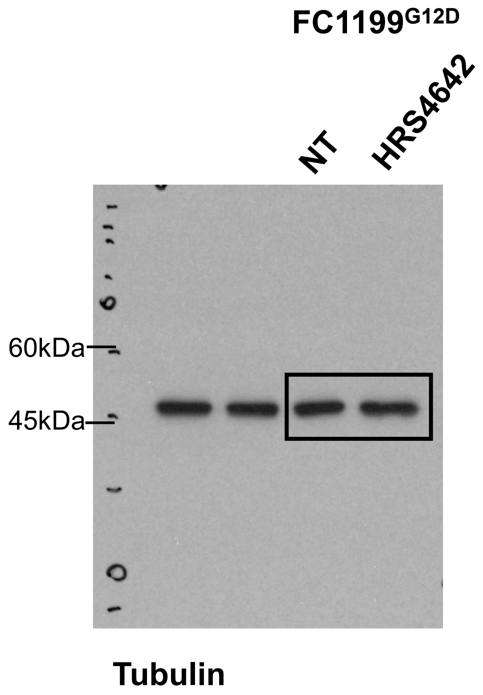

Original Western Blot Relative to Figure S1A

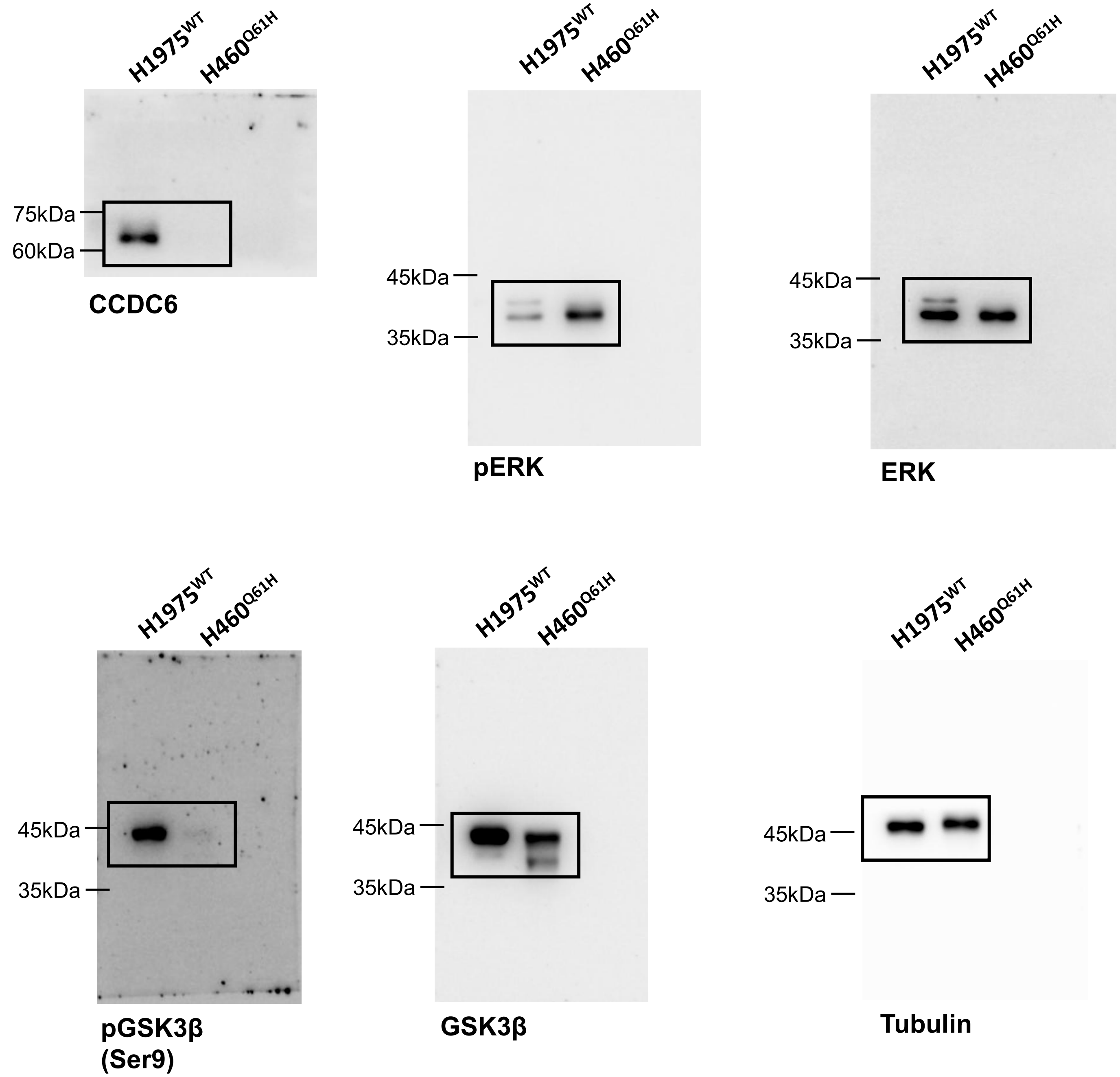

Original Western Blot Relative to Figure S1B

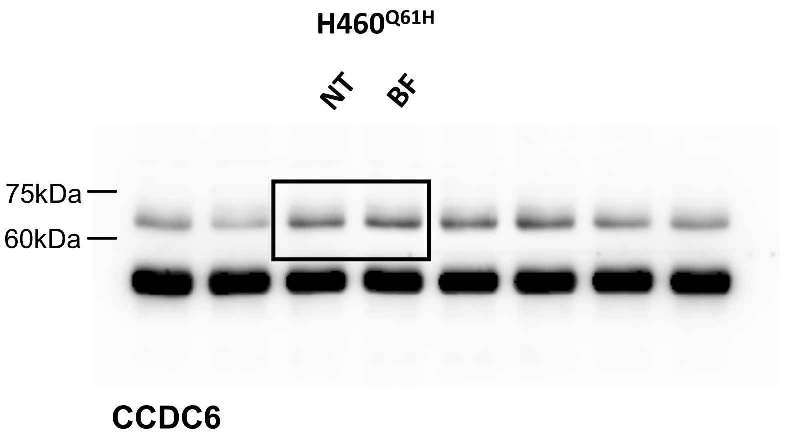

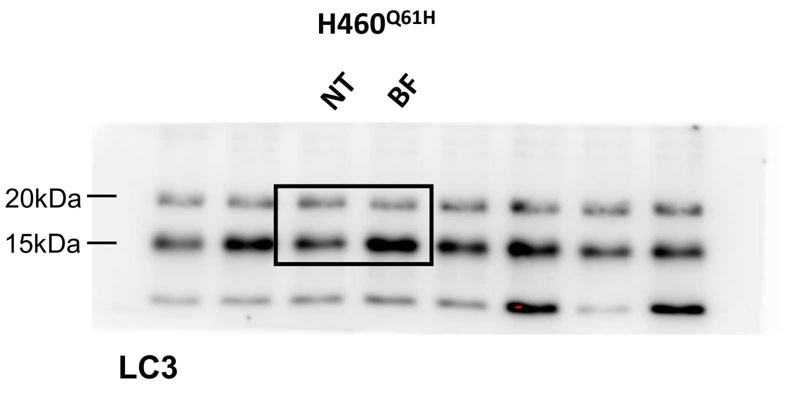

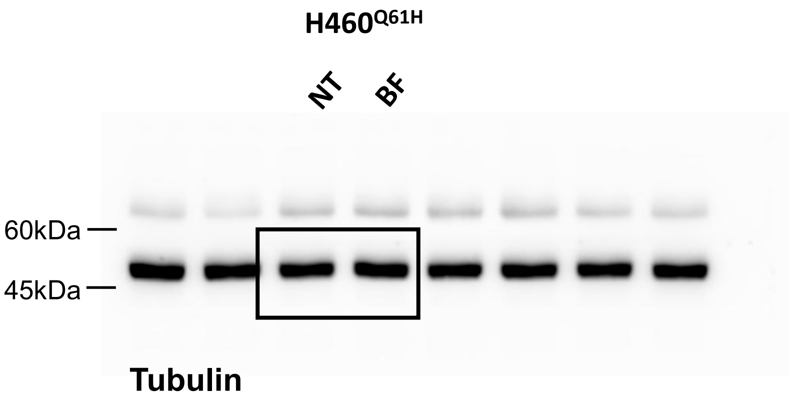

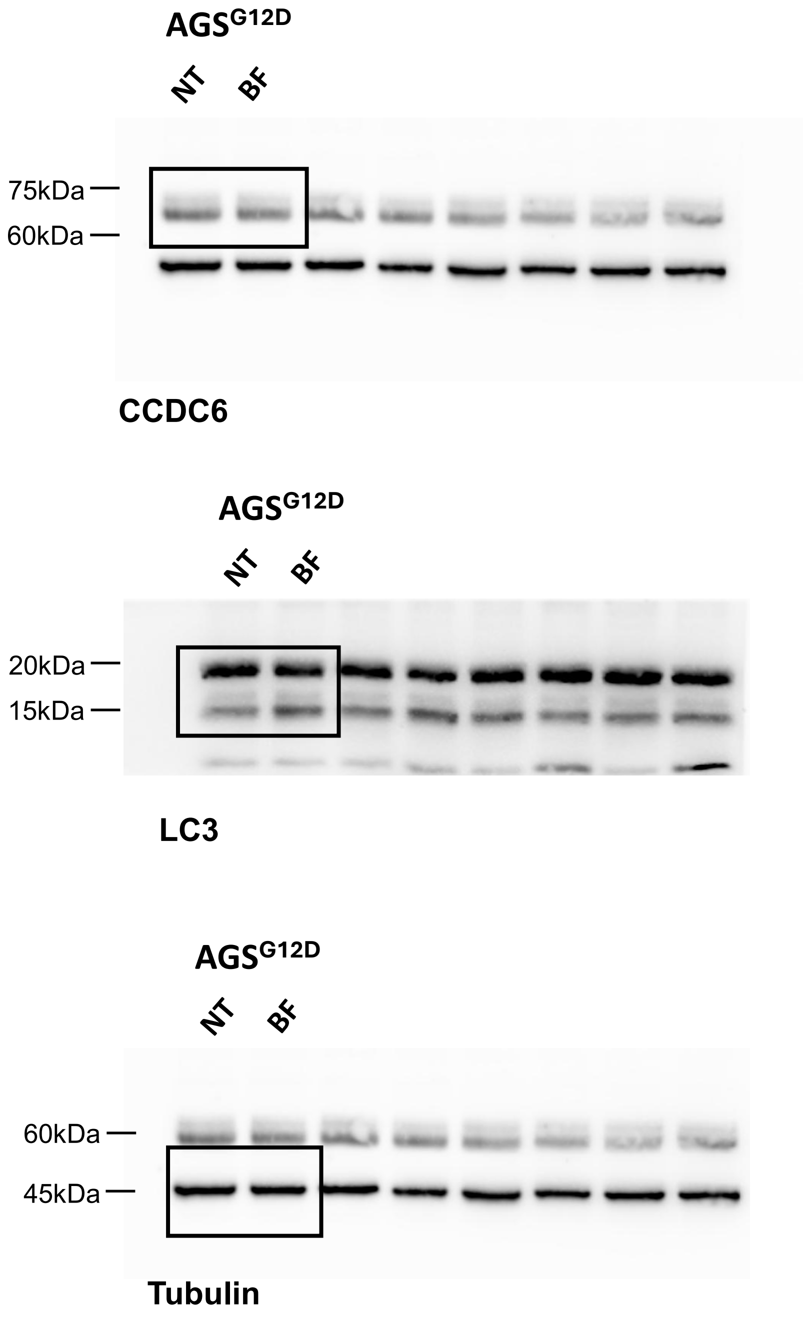

AGS^G12D
NT
BF
75kDa
60kDa
CCDC6
AGS^G12D
NT
BF
20kDa
15kDa
LC3
AGS^G12D
NT
BF
60kDa
45kDa
Tubulin

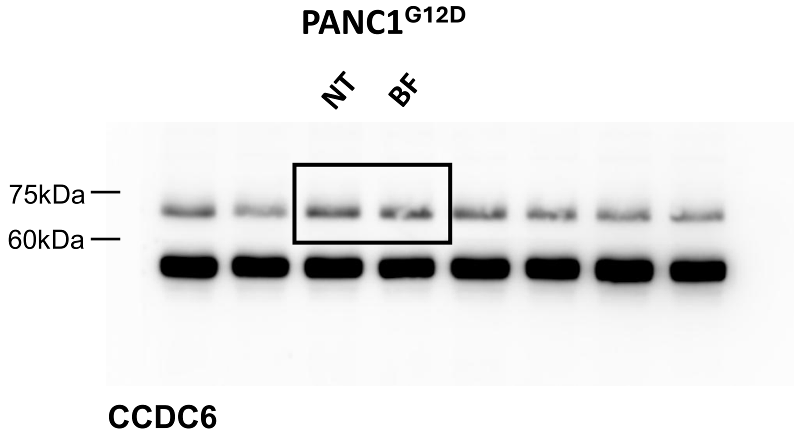
PANC1^{G12D}
NT
BF
75kDa
60kDa
CCDC6

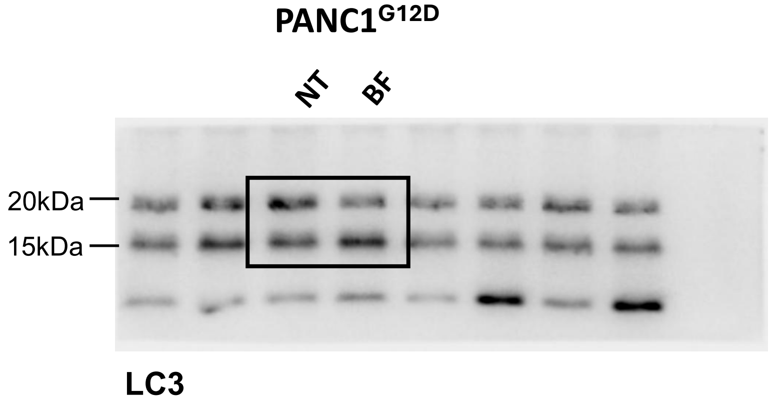
PANC1^{G12D}
NT
BF
20kDa
15kDa
LC3

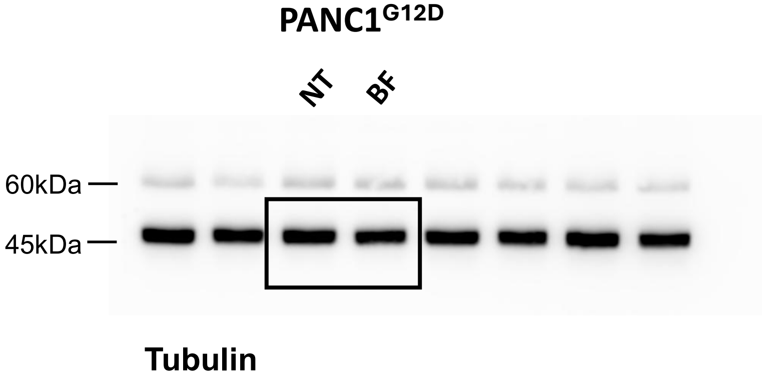
PANC1^{G12D}
NT
BF
60kDa
45kDa
Tubulin

Original Western Blot Relative to Figure S1C

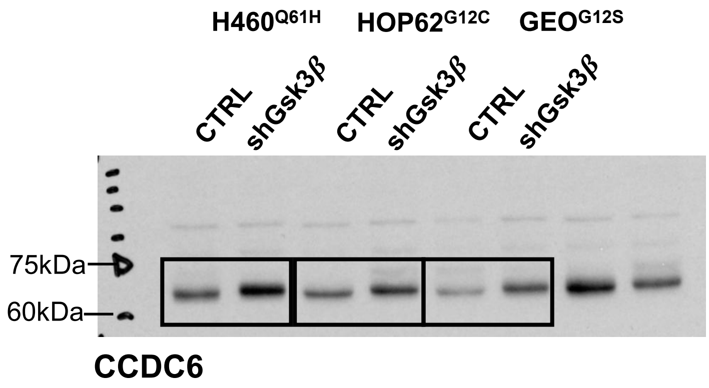

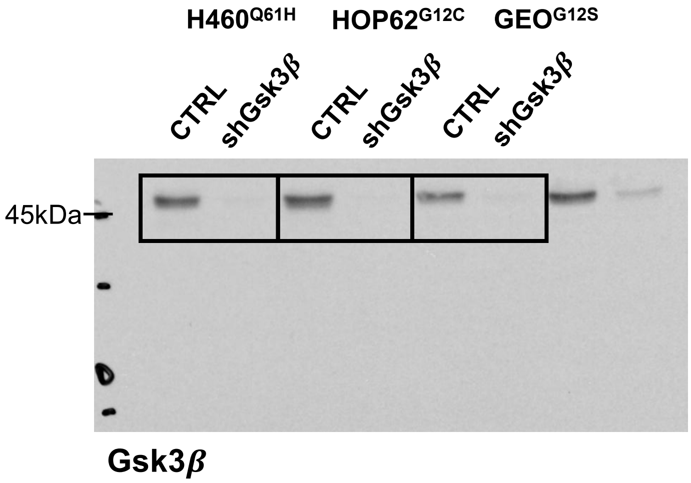

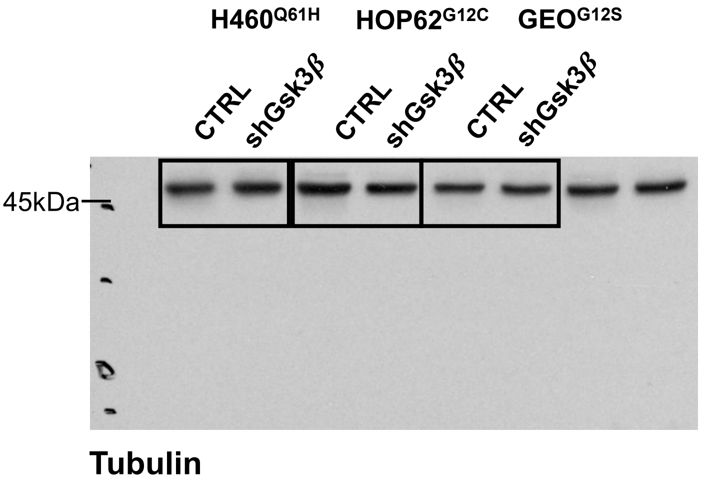

Original Western Blot Relative to Figure S2

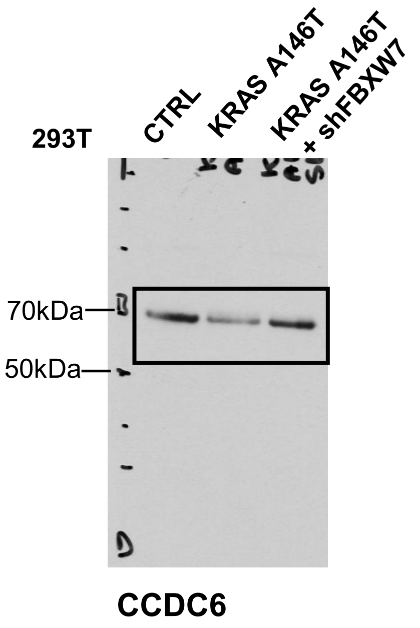

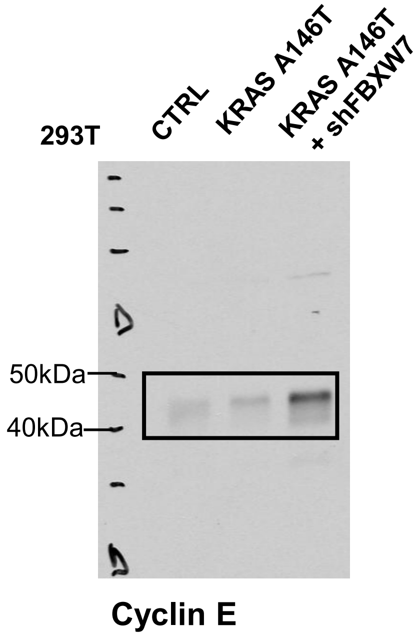

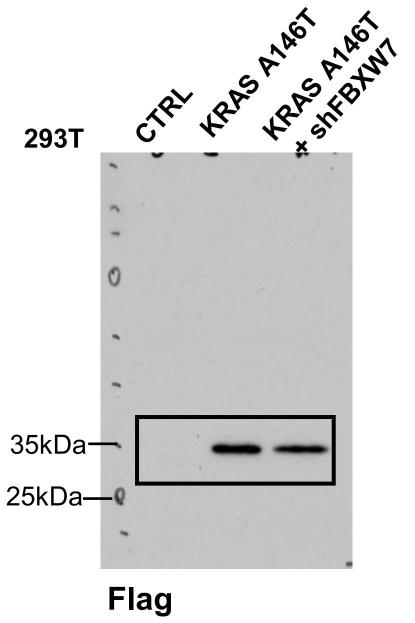

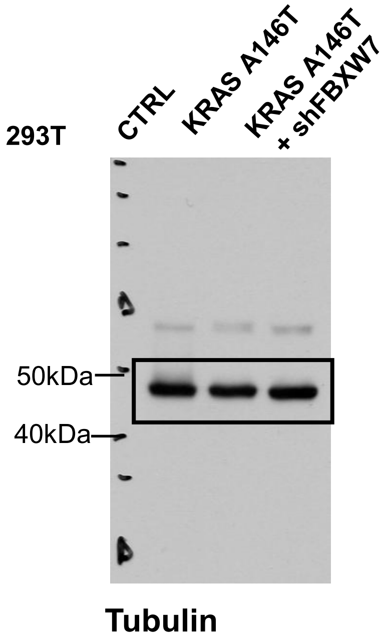

Supplement: Supplementary file 2 — Supplementary Material 2 [file 10495_2026_2400_MOESM2_ESM.pdf]
